# Structural Rearrangements of a Cobalt-Free Lithium-Rich Layered Oxide Cathode during Formation

**DOI:** 10.1021/acsaem.5c03511

**Published:** 2025-12-21

**Authors:** Matteo Busato, Mariarosaria Tuccillo, Arcangelo Celeste, Alessandro Tofoni, Laura Silvestri, Paola D’Angelo, Stefan A. Freunberger, Sergio Brutti

**Affiliations:** † Department of Chemistry, 9311 Sapienza University of Rome, P.le Aldo Moro 5, 00185 Rome, Italy; ‡ ALISTORE European Research Institute (ALISTORE ERI), Hub de l’Energie, 15 Rue Baudelocque, 80039 Amiens, France; § Department of Energy Technologies and Renewable Sources, ENEA, C.R. Casaccia, Via Anguillarese 301, 00123 Rome, Italy; ∥ Institute of Science and Technology Austria (ISTA), Am Campus 1, 3400 Klosterneuburg, Austria; ⊥ GISEL-Centro di Riferimento Nazionale per i Sistemi di Accumulo Elettrochimico di Energia, 50121 Florence, Italy; # Istituto dei Sistemi Complessi, Consiglio Nazionale delle Ricerche, P.le Aldo Moro 5, 00185 Rome, Italy

**Keywords:** lithium-rich layered oxides, Li-ion battery, cathode, Co-free, electrochemical performance, DFT, X-ray absorption spectroscopy

## Abstract

Formation during the first cycles of Li-rich layered oxide (LRLO) cathode materials consolidates the interphase and leads to structural changes that are decisive for long-term cyclability. However, the nature and effect of the changes are material-dependent and unknown for the important class of Co-free, Ni-poor LRLOs. Here, we analyze the processes during the tailored formation procedure of a typical class member, Li_1.28_Ni_0.15_Mn_0.57_O_2_, and demonstrate that it remarkably changes lattice composition and structure as a prerequisite for stable cycling. We combine electrochemistry, *operando* mass spectrometry, X-ray diffraction, and X-ray absorption spectroscopy with density functional theory simulations. Activation most prominently compresses the layer spacing along the *c*-axis and increases reversible structural breathing. The large capacity of ∼250 mAh g^–1^ originates from the Ni^2+^/Ni^4+^ and O^2–^/O^–^ redox couples. Electron exchange during O-redox is smeared over the entire anionic sublattice rather than localized on specific oxygen atomic sites. This redox mechanism is reversible without detrimental oxygen evolution, avoiding continued degradation common in conventional LRLOs. Sequential Ni- and O-redox during activation irreversibly distorts the coordination of the redox-inactive Mn^4+^ centers. This structural evolution of the MnO_6_ octahedra appears to enable the superior electrochemical performance of this LRLO phase. These findings define an activation pathway for the important class of Co-free, Ni-poor LRLOs, offering potential guidance for the rational design of high-performance, more sustainable cathode materials.

## Graphical abstract

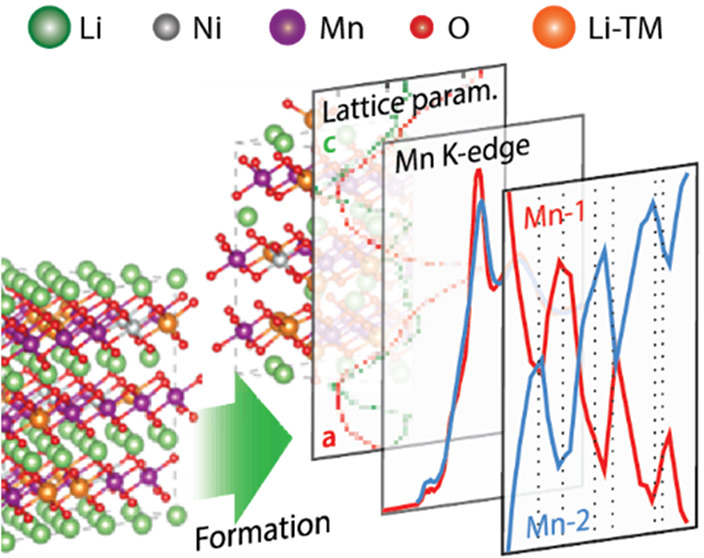

## Introduction

Secondary Li-ion batteries (LIBs) are a key-enabling technology to meet the goals of the “energy transition” from fossil to renewable energy sources and to implement the global shift from combustion engines to electric transport.^1–3^ This global need pushes LIBs market demand, requiring cheaper and more performing cells compared to the state-of-the-art. Innovations at the positive electrode are necessary to improve performance, costs, and environmental benignity.^4–6^

Li-rich layered oxides (LRLOs) offer a valuable alternative to current Li-stoichiometric transition metal oxides (TMOs) comprising nickel, manganese, and cobalt (NMCs). With Mn-rich TM blends, LRLOs have demonstrated superior reversible specific capacities beyond 200–250 mAh g^–1^.^1,7–9^ LRLOs have the general formula Li_1+*x*_M_1–*x*_O_2_, where M is typically a mixture of redox-active transition metals like Mn, Co, and Ni.^10^ They comprise stacked layers of MO_6_ octahedra, where overstoichiometric lithium partially occupies the TM sites.^1,8^ They crystallize in a complex lattice based on a monoclinic unit cell with partial ordering along the *a*- and *b*-axes and stacking faults along the *c*-axis: the extent of these distortions depends on the metal blend, lithium content, and the presence of oxygen vacancies.^7^ This extended defectivity can be qualitatively modeled either by a trigonal/rhombohedral lattice (LiCoO_2_ prototype, LCO) or a monoclinic structure (Li_2_MnO_3_ prototype): advanced structural prototypes, either rhombohedral or monoclinic, able to provide a realistic representation of this peculiar structure, have been discussed and validated by us in a recent publication.^11^ Besides pure performance, the development of new materials for electrochemical devices must satisfy mandatory requirements addressing costs and sustainability, issues that become increasingly more important day by day. In particular, the large use of cobalt poses ethical and political concerns, since cobalt commodities have reached limited availability, are expensive, and harmful to human health and the environment.^12^ These considerations are driving a “rush-for-cobalt-removal”, which, however, resulted in increasing market demand for nickel, which is next in line among the battery-related critical raw materials.^13,14^ In this framework, Co-poor and Co-free, Ni-poor LRLOs have already been reported in the literature,^14–19^ the most typical being Li_1.2_Mn_0.6_Ni_0.2_O_2_.^20,21^ They can deliver a reversible specific capacity above 200 mAh g^–1^ for hundreds of cycles at C/10, to be compared with 150–180 mAh g^–1^ of NMCs or Al-doped NMAs,^22,23^ and also showed excellent rate capability with 118 mAh g^–1^ at 2 C.^24^ To deliver this superb performance, low-Ni LRLOs require a controlled electrochemical formation protocol comprising a sequence of galvanostatic/potentiostatic steps.^24^ The effect of this formation appears to go beyond interface stabilization as in LiBs, which require, in general, formation cycles before the standard operation to stabilize interphases between the electrolyte and both electrodes, forming the so-called solid-electrolyte interphase (SEI) and cathode-electrolyte interphase (CEI), respectively. During formation under controlled electrochemical and temperature conditions, most of the inevitable, irreversible processes occur, enhancing the battery performance and limiting electrolyte consumption and lithium loss.^25,26^ Our preliminary investigations have shown that for the Ni-poor LRLOs, formation not only forms the interphases but, likely more importantly, leads to structural rearrangements.^24^ Critically, the mechanisms that govern the transition from the pristine, low-performing state to the stable, activated lattice-specifically the interplay between redox chemistry, bulk and local structure, and long-term cycling stability—are highly relevant for Co-free compositions. Their nature and effect are, however, unknown, while in-depth knowledge is required to understand electrochemical activity and stability, and for guiding the next generation of sustainable, high-energy cathode design.

Here, we analyze the structural evolution during the formation cycles of an overlithiated Co-free and Ni-poor LRLO cathode with the stoichiometry Li_1.28_Ni_0.15_Mn_0.57_O_2_. We used ex-situ and *operando* X-ray diffraction (XRD), *operando* X-ray absorption spectroscopy (XAS), online electrochemical mass spectrometry (OEMS), inductively coupled plasma optical emission spectroscopy (ICP-OES), and electrochemical techniques, supported by computational modeling based on the density functional theory (DFT) modified by the Hubbard model (+U). We find that formation not only forms a stable, low-impedance interface, but, more importantly, gradually and subtly transforms the environment of the redox-inactive Mn from the as-synthesized structure into one that allows for sustained reversible cycling of the Ni^2+^/Ni^4+^ and O^2–^/O^–^ redox couples.

## Materials and Methods

### Synthesis and Preliminary Characterization of Li_1.28_Ni_0.15_Mn_0.57_O_2_

Anhydrous LiNO_3_ was purchased from Carlo Erba reagents, while Mn­(NO_3_)_2_·4H_2_O, Ni­(NO_3_)_2_·6H_2_O, and sucrose from Merck. The LRLO Li_1.28_Ni_0.15_Mn_0.57_O_2_ was synthesized by solution combustion synthesis, followed by a thermal treatment in a muffle furnace, according to the procedure reported in refs 22–24 The crystal structure and morphology have been analyzed by XRD (Rigaku D/Max Ultima), Raman spectroscopy (Dilor Labram instrument equipped with a He–Ne laser), and scanning electron microscopy (ZEISS Gemini LEO 1530).

### Cell Assembly and Electrochemical Characterization

Electrodes were prepared by mixing the LRLO with Super P carbon and poly­(vinylidene fluoride) in a weight ratio of 80:10:10. A slurry was obtained by the addition of *N*-methyl-2-pyrrolidinone and cast on an aluminum foil to have a thickness of 200 μm. The coated aluminum foil was dried in an oven at ∼50 °C under vacuum for 3 h, calendared, and then disks with a diameter of 10 mm were cut. Electrodes were manufactured in a dry room at 20 °C and a dew point of −70 °C. As a final step, the disks were dried under vacuum at 110 °C overnight and then transferred to an Ar-filled glovebox with a moisture level below 1 ppm. Al-coated 2032 coin cells were assembled using the prepared disks as working electrode and lithium foil as counter electrode. Whatman GF/D paper soaked with LP30 electrolyte (1 M LiPF_6_ in ethylene carbonate:dimethyl carbonate 1:1 w/w) was used as a separator. Cell assembly was carried out in Ar-filled glove boxes (Iteco Eng SGS30, Mbraun Labstar) with oxygen and humidity levels below 1 ppm.

The lithium cells were electrochemically tested by galvanostatic cycling with a Maccor S4000 or MTI battery testing system. Charge/discharge tests were performed in the 2.0–4.8 V voltage range and using various current rates, with nominal 1C being 400 mA g^–1^ normalized by the active material mass. Long-lasting galvanostatic tests were performed on the cells before and after formation cycles.

Electrochemical impedance spectroscopy (EIS) tests were performed using an IVIUM Vertex apparatus using a sinusoidal voltage signal of Δ*V* = 10 mV in the 200 kHz–1 Hz frequency range. The composition of the pristine and post-mortem LRLO-based electrodes has been checked by electrochemical titration with a Li^+^-specific electrode (i.e., electrochemical Li^+^ titration, ELT) and ICP-OES on an ICPE-9820 (Shimadzu) system. Details about the ELT and ICP-OES tests are provided in the Supporting Information (SI).

OEMS measurements were performed to follow in *operando* conditions the possible CO_2_ and O_2_ gas evolution during formation. This setup consisted of a commercial quadrupole mass spectrometer (Hiden Analytical) with a turbomolecular pump (Edwards), backed by a membrane pump and an in-house-made leak inlet that samples the purge gas stream. The setup was calibrated for different gases (Ar, O_2_, CO_2_, H_2_, N_2_, and H_2_O) using calibration mixtures. All calibrations and quantifications were performed using an in-house software written in MATLAB. The purge gas system consists of a digital mass flow controller (Bronkhorst) and stainless steel or PEEK tubing.^27^ The electrochemical cell was a PAT-Cell-Gas (EL-CELL, Hamburg, Germany), which was kept at 30 °C inside a temperature chamber. The purge gas flow (Ar) was 0.700 mL min^–1^.

### Operando XRD

*Operando* XRD measurements were performed at the MCX beamline of Elettra-Sincrotrone Trieste (Italy). The employed cell was made with a 3D printer using polylactic acid with beryllium windows on both sides, as shown in Figure S4a. A picture of the experimental setup at the beamline is shown in Figure S4b. The experiment was carried out using a wavelength of 1.03 Å (12 keV) in the 2θ range 10–35° using a MarCCD-SX-165 2D detector. Each diffractogram was recorded with an acquisition time of 20 s during the first two formation cycles, giving a total of more than 200 diffractograms.

Diffraction patterns were integrated from the 2D images and analyzed using the GSAS-II software.^28^ Simulated lattice, assuming a rhombohedral LCO prototype, have been matched to the experimental XRD along the entire *operando* experiment by using the Rietveld refinement routines embedded in the GSAS-II code.

### Operando XAS

XAS measurements at the Ni and Mn K-edges were performed in transmission mode at the XAFS beamline of Elettra-Sincrotrone Trieste.^29^ A focusing double-crystal Si(111) monochromator was employed, while the storage ring was operating at 2 GeV and the beam current was 200 mA. The polylactic acid 3D-printed cell with beryllium windows on both sides (Figure S4a) was aligned between the first and the second ionization chamber. A picture of the experimental setup is shown in Figure S4c. XAS spectra were recorded during battery operation in the first formation cycle and part of the second one, alternating every *ca*. 30 min between the Ni and Mn K-edges. Data could not be collected on the entire second cycle due to the end of the allocated beamtime. Ni and Mn metal foils were used for energy calibration. In the extended X-ray absorption fine structure (EXAFS) region, the data were acquired up to *k* = 11 and 12 Å^–1^ for Ni and Mn, respectively. XAS spectra were also collected with the same experimental conditions for solid NiO, LiNiO_2_, Mn_2_O_3_, and MnO_2_ as references.

The complete *operando* XAS data sets at both the Ni and Mn K-edge were analyzed by combining principal component analysis (PCA) and multivariate curve resolution (MCR) methods with the PyFitit code.^30^ The EXAFS region of the MCR-reconstructed absorption spectra of the pure components was analyzed with the GNXAS code.^31,32^ Advanced theoretical calculations of Mn K-edge X-ray absorption near edge structure (XANES) spectra have been carried out with the finite differences method near-edge structure (FDMNES) program, a DFT code able to calculate XAS spectra within both the full multiple-scattering (MS) and finite differences method (FDM) theoretical frameworks.^33,34^ XANES spectra were calculated starting from the DFT + *U* optimized structures for the pristine and partially delithiated electrode material (*vide infra*).

More details about the PCA-MCR, EXAFS analysis, and theoretical XANES calculations are provided in the SI.

### DFT + *U* Simulations

DFT calculations were carried out on a simulated LRLO structural model constituted by a 5 × 4 × 1 supercell built from the *R*3̅*m* nonprimitive hexagonal unit cell of the prototype LiCoO_2_. To simulate the pristine (reduced) and the charged (oxidized) states, two different stoichiometries have been considered for the DFT modeling, both starting from the Li_1.28–x_Ni_0.15_Mn_0.57_O_2_ formula and assuming *x* = 0 (fully lithiated LRLO, FL-LRLO) and *x* = 0.67 (partially delithiated LRLO, PL-LRLO).^23,24^ Additional details about the building of the supercells are given in the SI.

All the calculations have been performed with spin-polarized Kohn–Sham DFT with projector-augmented wave potentials and plane wave basis sets, using the periodic supercell approach as implemented in the 5.4.1 version of the Vienna Ab initio Simulation Package (VASP).^35^ A kinetic energy cutoff of 520 eV was employed, and the Brillouin zone was sampled at the Γ-point. We applied the generalized-gradient approximation (GGA) within the exchange-correlation functional by Perdew, Burke, and Ernzenhof (PBE).^36^ The rotationally invariant DFT + *U* approach^37^ was employed to amend the self-interaction error, with a *U*–*J* value of 4 eV for the Ni and Mn d electrons. Atomic positions and lattice parameters have been optimized separately by minimizing total energy and forces without any symmetry constraint until the residual force on each atom was <0.01 eV Å^–1^.

## Results and Discussion

The preliminary physicochemical characterization of the pristine LRLO is shown in the Supporting Information (Figure S1), where the XRD pattern, the Raman spectra, and the SE micrographies are reported. The pristine LRLO material consisted of well-formed, very homogeneous in shape, and size prismatic crystallites (likely octahedral) of approximately 100 nm in diameter. The XRD pattern confirms the excellent crystallization of the expected trigonal/rhombohedral lattice (LiCoO_2_ prototype, LCO) with partial ordering in the TM layer, shown by the expected appearance of the weak peaks in the 20–30° 2Θ range. The Raman spectrum confirms the absence of spinel-like contamination on the material surface and highlights multiple bands in the 350–700 cm^–1^ range due to the various TM-O vibrational modes from the hexagonal/monoclinic LRLO lattice.

### Interphase Evolution during Formation

The beneficial impact of the electrochemical activation protocol on the subsequent performance during cycling at a constant C-rate is shown in Figure 1, comparing the capacity retention and electrode potential upon cycling of cells undergoing formation or not. Despite a comparable specific capacity of ∼250 mAh g^–1^ in the first cycle, the performance is remarkably improved upon cycling for the cell initially submitted to the formation procedure (Figure 1a). The capacity retention at, e.g., cycle 30, improved from 82% without formation to 98% with formation (Figure 1b), discharge capacity by 35 mAh g^–1^ from 210 to 245 mAh g^–1^, and the mean energy density by 119 mWh g^–1^ from 745 to 864 mWh g^–1^. This enormously improved reversible performance parallels a slightly altered potential profile (Figure 1c,d). For example, in cycle 30, the mean charge potentials grew from 3.85 to 3.90 V vs Li^+^/Li without and with formation, respectively, and discharge potentials remained constant at 3.20 V for both cells. Importantly, the activation appears required for very stable cycling, as shown in Figure 1a,b, already demonstrated in refs 22–24 , and in Figure S2.

**Figure 1:**
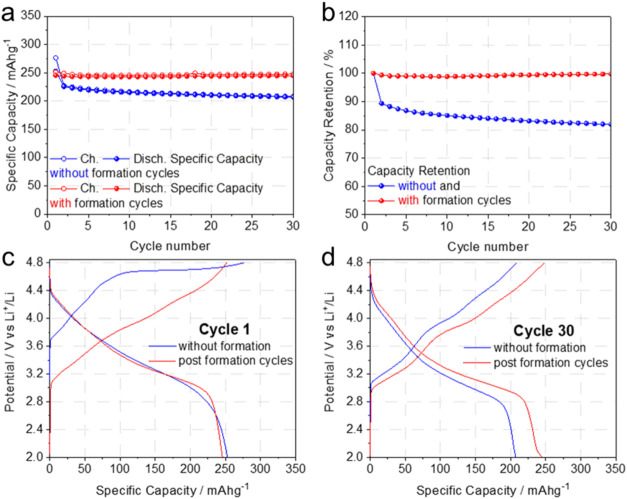
Performance from cycle 1 (without formation) or postformation in lithium cells formulated with the LRLO electrodes. (a) Comparison of the specific capacities at C/10, (b) discharge capacity retentions at C/10, (c) comparison between the LRLO electrode potentials in the first cycle at C/10, and (d) comparison between the LRLO electrode potentials at C/10 at cycle 30 (1C = 377 mA g^–1^).

It is important to recall that the formation protocol consisted of three constant current–constant voltage (CC–CV) steps (named Stage A, B, and C) performed at C/10, C/5, and 1C for two cycles each, as follows:

- **Stage A**: two galvanostatic cycles at C/10 with a potentiostatic hold at 4.8 V to a current cutoff of C/20, followed by a rest of 1 h.
- **Stage B**: two galvanostatic cycles at C/5 with a potentiostatic hold at 4.8 V to a current cutoff of C/20, followed by a rest of 1 h.
- **Stage C**: two final cycles at 1C with a potentiostatic hold at 4.8 V to a current cutoff of C/20.

The evolution of the cathode potential profiles during the formation cycles is shown in Figure 2a. While the first charge profile shows the typical sloping rise to 4.4 V, followed by a long pseudoplateau at 4.5–4.7 V vs Li^+^/Li, the following cycles show nearly featureless charge profiles. In contrast, the discharge profiles are almost unaltered upon cycling, changing only as the current increases. The formation procedure shows an overall Coulombic efficiency (CE) of 90.4% and a cumulative irreversible capacity loss of 149 mAh g^–1^. This suggests limited parasitic chemistry to occur, mainly in the first C/10 cycle (CE 78.4%) and in the first 1 C cycle (CE 81.9%).

**Figure 2:**
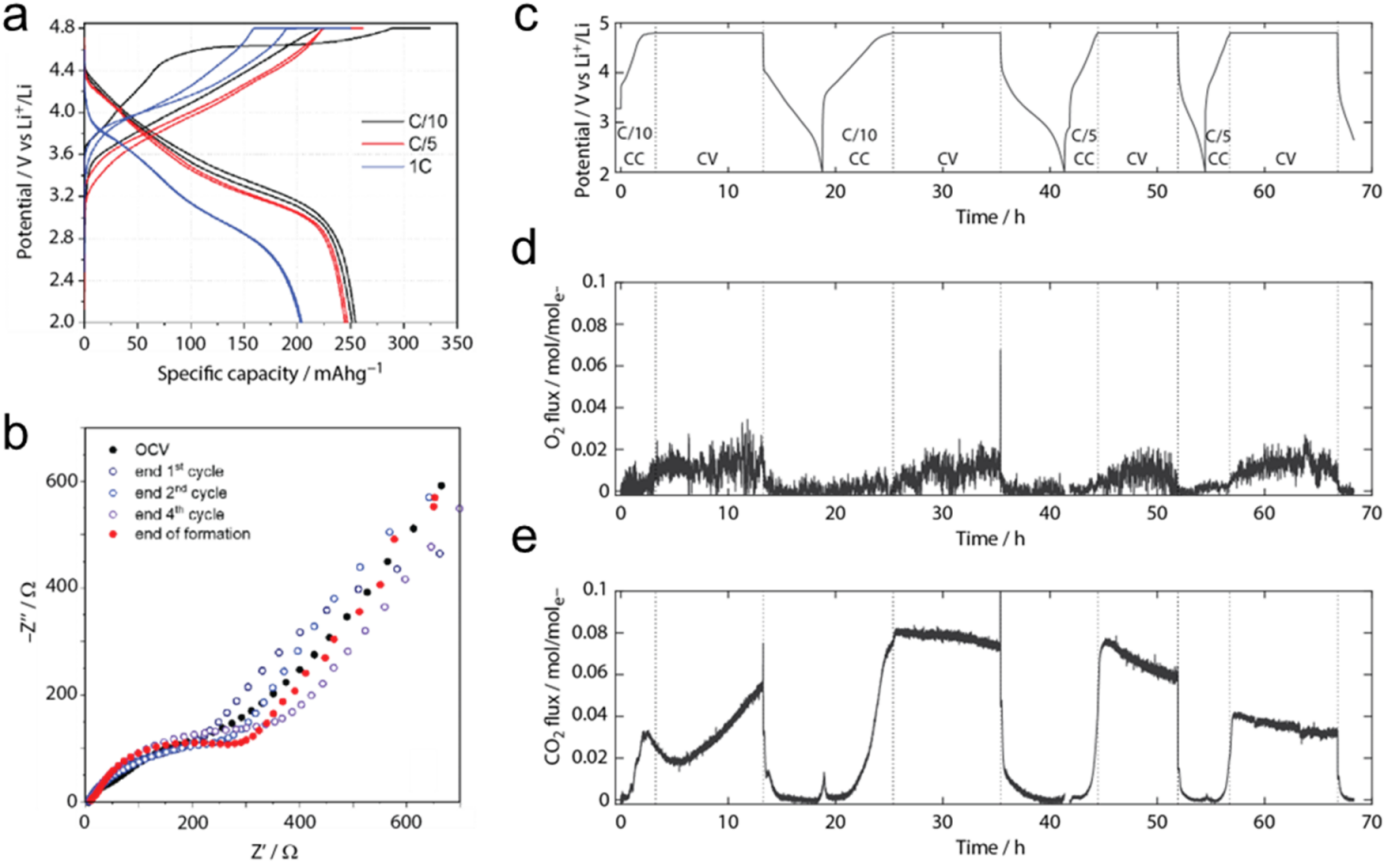
Analysis of the electrochemical response of LRLO electrodes in lithium cells during the formation protocol: (a) Potential profiles, (b) EIS responses in the lithiated states (discharged electrodes), (c) potential profiles during the OEMS experiment, (d) evolved O_2_, and (e) CO_2_ flux normalized to electron flux as measured by OEMS.

The EIS profiles of the LRLO electrodes in their lithiated states during the formation (Figure 2b) show only minor changes. Qualitatively, in all cases, two depressed, largely overlapping semicircles can be identified, with time constant in the μs and ms range, respectively, followed by a linear Warburg diffusion slope. This EIS shape likely implies a dielectric response originating from an SEI, a charge transfer reaction, and solid-state diffusion. The equivalent circuit analysis performed using the model shown in Figure S3a suggests a relatively constant SEI resistance *R*_SEI_ after the second cycle, approaching ∼12–21 Ω (Figure S3b). In contrast, the charge transfer resistance *R*_CT_ increases slightly from 150 to ∼200 Ω. EIS suggests a progressive change in the (de)­lithiation kinetics of the LRLO lattice during formation. The attained stable SEI resistance suggests a stable thickness and composition and, therefore, a stable electrolyte/electrode interface.

The consolidation of the interface occurs in parallel with the release of CO_2_ and small amounts of O_2_, as shown by OEMS, with gas fluxes normalized to electron fluxes (Figure 2c–e). During the first charge, CO_2_ evolves from ∼3.8 V, indicating Li_2_CO_3_ as the source^38,39^ and first decaying and then again increasing rate during the 4.8 V hold. In the following cycles, the onset of CO_2_ release is at ∼4 V, with decaying rates during the holds. The latter is in accord with a consolidating SEI. A small amount of O_2_ (∼1 O_2_/100 e^–^) is equally seen during the holds, suggesting at least some of the O in the CO_2_ stems from lattice O. The gas release is in accord with a certain amount of parasitic charge and hence the observed irreversible capacity losses during the formation cycles. Outgassing of CO_2_ and O_2_ is typical for high-voltage positive electrode materials like LRLOs due to the oxidation of electrolyte constituents, residual Li_2_CO_3_, or gassing of the material. Previously, studies on related LRLOs such as Li_1.2_Ni_0.13_Co_0.13_Mn_0.54_O_2_ and Li_1.2_Mn_0.6_Ni_0.2_O_2_ reported typical O_2_ and CO_2_ evolution patterns as reported in refs 40–42 It starts with minor onsets of CO_2_ evolution at ∼4 to 4.2 V and then a first CO_2_ burst at the start of the O-redox plateau at 4.5 V, followed by another CO_2_ burst at the end of the plateau, where the voltage turns up toward the cutoff potential. O_2_ has only been seen at the latter burst, but isotopic ^18^O labeling of the LRLO has shown that both the CO_2_ and O_2_ contained lattice oxygen. CO_2_ seen in our case may hence stem from lattice oxygen. These studies used, however, different Co-free LRLOs with the stoichiometry Li_1.2_Mn_0.6_Ni_0.2_O_2_ for which pronounced changes in the manganese environment and localized holes on oxygen were found, different from what we see here, as discussed in the following sections.

The Li_1.28_Ni_0.15_Mn_0.57_O_2_ stoichiometry used here is a strongly p-doped variant of Li_1.2_Mn_0.6_Ni_0.2_O_2_, as Li^+^ acts as an acceptor dopant while it replaces Mn^4+^ and Ni^2+^. Therefore, the nominal oxidation state of Ni is +3 in Li_1.28_Ni_0.15_Mn_0.57_O_2_, whereas it is +2 in Li_1.2_Mn_0.6_Ni_0.2_O_2_. This difference has a remarkable impact on the redox mechanism as it induces activation of the O^2–^/O^–^ redox couple, starting from a nominal stoichiometry Li_1.13_Ni_0.15_Mn_0.57_O_2_ as compared to Li_0.8_Mn_0.6_Ni_0.2_O_2_ in the mentioned studies.^40–42^ Different from them, the Mn environment changes only mildly, and the holes on oxygen are not localized but form a diffuse electron-poor anion sublattice. We speculate that this difference leads to an altered oxygen chemical potential in the p-doped LRLO lattice and, therefore, to an upshift of the onset potential for the disproportionation of peroxide moieties in the delithiated LRLO, the latter being the primary source of molecular oxygen. One of the most prominent changes in the LRLO lattice during the formation is the degree of lithiation at the end of each formation stage. The ICP-OES and ELT analyses of Li contents measured post-mortem after the three stages of formation are summarized in Table 1, in comparison to the nominal Li content based on capacity. After stages A and B of formation, the actual Li content in the LRLO is smaller by ∼5% and 27% as compared to the values expected from the capacity. These differences prove the occurrence of parasitic electrochemistry, in accord with CO_2_ and O_2_ release and irreversible capacity losses. Remarkably, at the last stage of formation (stage C), the LRLO electrode can reincorporate some of the Li^+^ ions lost earlier despite the faster 1C rate compared to C/10 and C/5 in the previous stages. Accordingly, Li^+^ extraction at C/10 and C/5 appears to be more facile compared to insertion, given that, at the end of stage B, the LRLO is remarkably Li-poor, reaching only Li_0.71_Mn_0.57_Ni_0.15_O_2_. Surprisingly, upon the following Li^+^ extraction at 1C in stage C, 249 mAh g^–1^ can be extracted despite this phase formally only allowing for a theoretical capacity of 234 mAh g^–1^. This surprising finding has been replicated 3-fold with different cells, using different synthesis batches of the same LRLO stoichiometry. Thus, this peculiarity is a further clue for parasitic chemistries upon charge that do not extract Li^+^ ions. It furthermore highlights that this formation procedure allows to electrochemically extract almost all Li^+^ ions from the LRLO lattice, even those present in the TM layer. This interpretation implies the formation of a large void concentration in the TM layer, likely inducing structural rearrangements. Consequently, the activated LRLO lattice can more easily intercalate Li^+^ upon discharge, allowing the partial recovery of the Li content.

**Table 1:** Composition of the LRLO Electrode Li_*x*_Mn_*y*_Ni_*z*_O_2_ at Various Stages of Formation^a^

stage	Li*_x_* ^b^	Li*_x_* ^c^	Mn*_y_* ^d^	Ni*_z_* ^e^
pristine	1.28(1)	1.28	0.57(2)	0.15(1)
stage A	1.00(4)	1.05	0.57(2)	0.15(1)
stage A + B	0.71(2)	0.98	0.54(2)	0.15(1)
stage A + B + C	0.88(5)	0.82	0.54(2)	0.14(1)

a Standard deviation in parentheses.

b From ICP-OES and ELT.

c Nominal content from the electrochemical data.

d From ICP-OES.

e From ICP-OES of pristine material.

Concerning the TM content in the LRLO lattice at the end of stage B, as well as at the end of the entire protocol, a slight loss of Mn and Ni is observed. A slight TM depletion induced by dissolution from the crystallite surfaces is expected due to the possible disproportionation of TM^3+^ centers to form soluble TM^2+^ ions.^43^

### *Operando* XRD: Changes in the LRLO Bulk during Formation

Two *operando* XRD tests have been carried out to probe for possible permanent structural rearrangements in the LRLO lattice during the electrochemical formation. First, the first two formation cycles starting from pristine LRLO, and second, two cycles of a fully activated LRLO, which have already passed all formation stages. The complete data sets are shown in Figures S5 and S6. All XRD patterns were Rietveld refined to obtain the evolution of the hexagonal lattice parameters of the LRLO (Figure 3).

**Figure 3:**
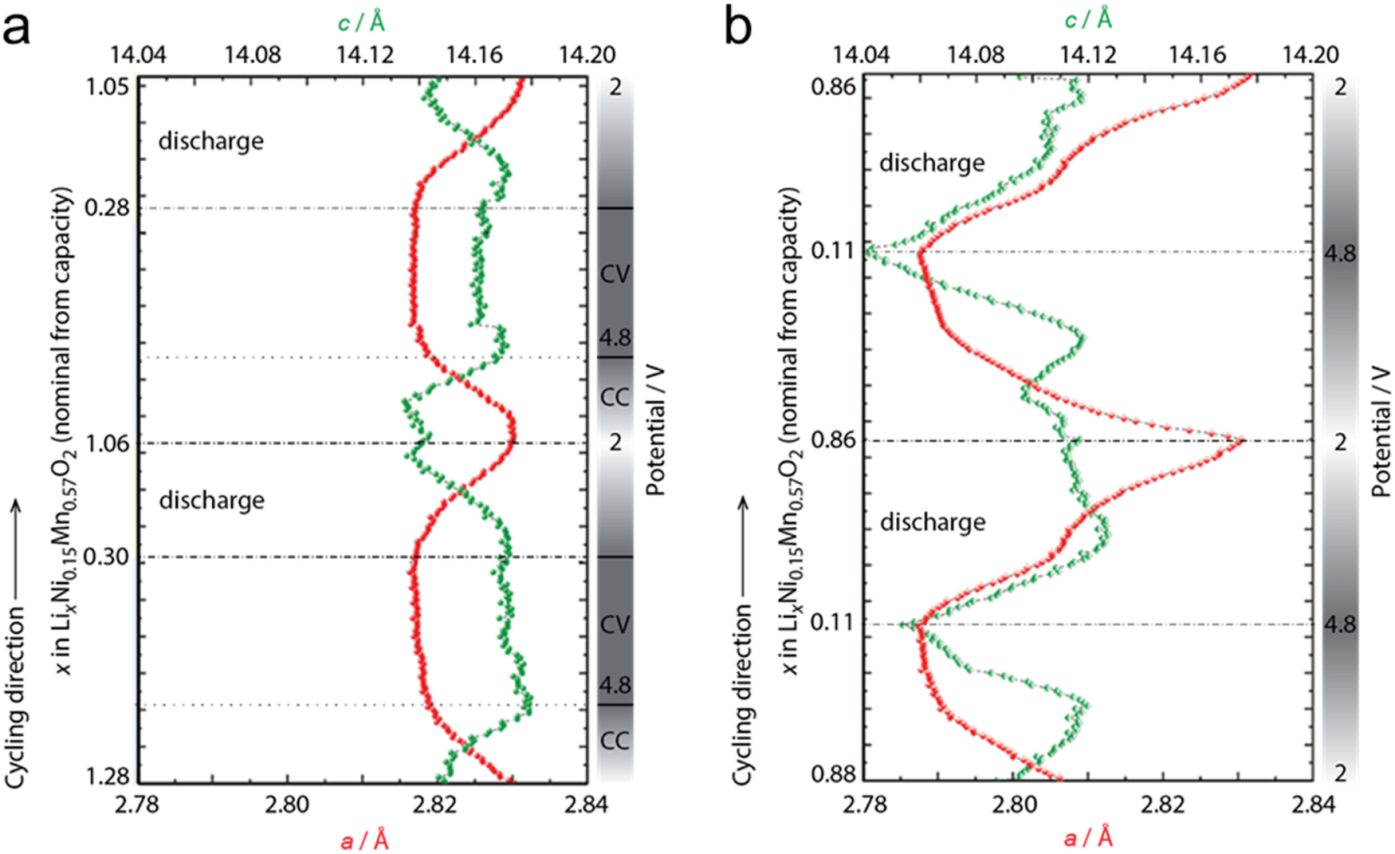
LRLO hexagonal lattice parameters (LCO prototype) estimated by Rietveld refinement of the *operando* XRD data. (a) The first two cycles starting from pristine LRLO. Cycling at C/10 between 2.0 and 4.8 V and a hold at 4.8 V to a current cutoff of C/20. (b) Two cycles of a fully formed LRLO electrode. Cycling at C/10 between 2.0 and 4.8 V. Each XRD acquisition time was 20 s. The left axis shows the Li^+^ stoichiometry based on capacity. XRD data and voltage versus time are shown in Figures S5 and S6.

The hexagonal crystal of the pristine LRLO evolves in two steps during stage A of formation (Figure 3a). In the first part of delithiation (charge), the *a*-axis is compressed with concurrent expansion of the *c*-axis. The shrinking of the *a*-axis likely results from the oxidation of Ni^3+^ to Ni^4+^, which has a much smaller Shannon radius.^44^ All TMs in the LRLO occupy a planar hexagonal honeycomb-like layer orthogonal to the *c*-axis, where six other TM/Li^+^ ions surround each TM. Therefore, the shrinking size of Ni ions upon their electrochemical oxidation shortens the Ni–Ni, Ni–Mn, and Ni–Li bond lengths, and hence the *a*- (and *b*-) axis. On the contrary, the expanding *c*-axis likely results from the weaker bonding between the adjacent O and Li layers, both stacked in the *c*-direction, while the Li^+^ ions are electrochemically removed from the lattice upon charge.

In the second step, corresponding with the 4.8 V hold, both the *a*- and *c*-axes remain constant, likely due to various balanced phenomena. First, the continued removal of Li^+^ ions from the lattice, shortening the *c*-axis, which is normal to the planar layers. Second, the extraction of electrons from electronic states, which are mainly delocalized over the anion sublattice. This redox reaction, typically referred to as the O^2–^/O^–^ oxidation, leads to a mean oxidation state of oxygen >−2, thus weakening the TM-O bonds. The net effect is a growing lattice volume as the TMO_6_ and LiO_6_ octahedra are neither aligned to the *ab*-planes nor the *c*-direction. Another possible effect is also related to the reduction of the mean oxidation state of oxygen anions: in fact, this phenomenon unavoidably reduces the O–O repulsion across the almost planar oxygen layers orthogonal to the stacking direction, thus shrinking the *a*- (and *b*-) axis. Upon discharge, remarkably, an inverse lattice modification occurs, with initially little change, followed by expansion/contraction of the *a*-/*c*- axes to nearly recover their initial values.

The hexagonal crystal of the activated LRLO electrode in two consecutive galvanostatic cycles at C/10 recorded after a complete formation process (Figure 3b) shows a more nuanced structural evolution on charge and discharge, which qualitatively follows similar trends compared to those observed in stage A of formation. However, the expansion and shrinking of *a* and *c* cell parameters are much more significant (∼2.8% and 0.5%, respectively), thus suggesting a more flexible lattice able to reversibly accommodate significant structural breathing while Li^+^ ions are (de)­inserted. Furthermore, while the *a*- (and *b*-) axis in the fully lithiated (discharged) state matches the size of the pristine lattice, the *c*-axis is compressed in both the lithiated and delithiated states, compared to the original size before formation.

### *Operando* XAS: Irreversible Local Structural Rearrangement during Formation

To obtain further insights into the electronic and structural modifications of the LRLO material during formation, we performed *operando* XAS measurements during stage A and part of stage B of the activation protocol. A chemometric approach based on PCA–MCR analysis of the *operando* data sets was used to decouple and quantify the evolution of the TM species. The XANES region of the pure component spectra identified for Ni is shown in Figure 4a and compared with those of NiO and LiNiO_2_ standards. The K-edge energy position E_0_ allows inspection of the Ni valence state. To extract E_0_, due to the presence of different contributions, the edge region was fitted with a Lorentzian function accounting for the pre-edge peak inner atomic transition at about 8342–8343 eV, plus an arctangent function to model the K-edge electronic transition (Figure S8). The edge energy extracted for pure component 1 (8344.6 eV, Figure S8a) almost matches that obtained for the NiO standard (8344.4 eV, Figure S8b), taken as the reference for the Ni^2+^ state, and lies at lower energy than LiNiO_2_ (8345.5 eV, Figure S8c), the reference for Ni^3+^. The spectrum of pure component 2 shows substantially higher energy than LiNiO_2_, thus strongly suggesting the formation of a Ni^4+^ valence. Note that XAS data could not be collected on a reference sample with a formal Ni^4+^ state due to the instability of its compounds. Nevertheless, the two independent components are in accord with a Ni^2+^/Ni^4+^ redox activity. The pristine LRLO starts with component concentrations at ∼70:30 ratio (Figure 4d), in agreement with an overall Ni^3+^ state. During the first delithiation, the fraction of component 1 decreases and remains at a minimum toward the end of the first charge (EOC1, see Figure 4c for the potential cycling curve), with component 2 mirroring this behavior and reaching a ∼10:90 ratio. Nearly constant Ni^2+^/Ni^4+^ fractions for part of the profile suggest O-redox dominating in these regions. Notably, the small reduction in component 2 during the high-potential hold, mirrored by a slight increase in component 1, may reflect reductive coupling—i.e., Ni^4+^→Ni^3+^ reduction at high potentials - an unusual behavior previously reported by XANES for other Co-free Li-rich cathode materials during delithiation.^45^ During discharge, the overall trend is inverted, with component 1 (2) reaching its maximum (minimum) at the end of the first discharge (EOD1). This trend is nicely maintained in the second cycle at C/10 and in stage B up to EOD3, suggesting a remarkable reversibility of the Ni redox activity. Note that a scree plot statistical test, assessing the significance of individual components beyond the noise level (Figure S7a), suggests a third component to be of a certain significance. However, the reconstructed spectra of components 1 and 3 are very similar (Figure S9a), and their fractions evolve similarly during cycling (Figure S9c), resulting only in moderately increased fit quality when the third component is included.

**Figure 4:**
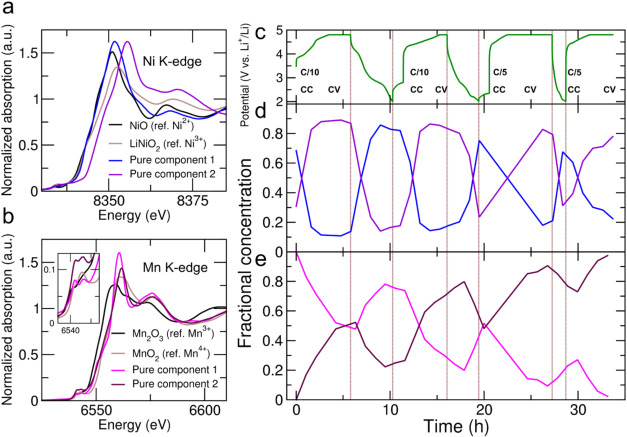
XANES spectra of MCR analysis-derived pure spectral components compared with the reference compounds for (a) Ni and (b) Mn K-edge (inset: magnification of the prepeak region corresponding to the 1s → 3d/4p transitions). (c) Evolution of potential profiles during the first two formation cycles. Evolution of fractional concentration of the two major components derived from the (d) Ni and (e) Mn K-edge, respectively. Vertical lines are added to indicate the different charge states. CC and CV indicated constant current and voltage phases during charging.

For the Mn K-edge, the scree plot shows two dominant components, while additional components are less clearly distinguishable in significance for fit quality (Figure S7b). The reconstructed spectra (Figure 4b) show less evident differences in terms of the edge position as compared to Ni, consistent with little participation of Mn in the redox activity. The edge energy position of these spectra is close to MnO_2_, the reference for Mn^4+^, while the XANES spectrum of the Mn_2_O_3_ standard, the reference for Mn^3+^, is found at markedly lower energies. The evolution of the concentration profiles shows that, during cycling, component 1 (2) reaches local minima (maxima) at full charge (discharge) with a trend of component 2 gradually becoming more dominant over repeated cycles (Figure 4e). Hence, unlike the Ni case, the starting concentration of component 1 never totally recovers, and component 2 gradually takes over. This trend highlights gradual irreversible structural rearrangements occurring in the local coordination environment of the Mn^4+^ ions. This is further supported by inspection of the pre-edge region (inset of Figure 4b), which records the dipole-forbidden 1s → 3d inner atomic transition, partially allowed when Mn 3d/4p orbital hybridization occurs due to distortions around a centrosymmetric Mn center.^46–49^ In pure component 2, the intensity of both pre-edge contributions increases compared to pure component 1, consistently with enhanced deformations in the MnO_6_ octahedra. Increasing distortions of octahedral Mn sites observed by XAS were previously discussed as the structural changes affecting Mn during cell cycling for other Li-rich layered formulations.^40,50–52^

EXAFS analysis was carried out to obtain more quantitative information about the local structure around the TM centers. It is important to note that the MCR analysis was applied to the full energy range of the experimental spectra, encompassing both the XANES and EXAFS regions. In this way, we could perform the EXAFS analysis directly on the MCR-derived pure components. The purpose of this approach, as opposed to conventional fitting of individual time-resolved spectra, is to decouple coexisting species and isolate their pure structural properties. Further details on the single- and multiple-scattering paths used in the EXAFS analysis are provided in the SI and Figure S10.

The result of the fitting is shown in Figure 5, where the theoretical signals and the total theoretical contributions are compared with the experimental data and the residuals. The theoretical and experimental data agree excellently in all cases, as also seen in the Fourier transform (FT) spectra at the bottom of Figure 5. The optimized structural parameters for the Ni­(Mn)-O and Ni­(Mn)-TM^first^ two-body distributions are listed in Table 2, the latter referring to the Ni and Mn scattering centers closest to the photoabsorber. The structural parameters for the Ni­(Mn)-TM^second^ and Ni­(Mn)-TM^third^ two-body distributions are listed in Table S1. The striking outcome is that the average Ni–O distance massively contracts from 2.04(2) to 1.89(2) Å as component 1 is converted into component 2. This contraction of the Ni–O bonds in the NiO_6_ octahedra is consistent with the shrinking ionic radii passing from the Ni^2+^ to the Ni^4+^ state and agrees with previous EXAFS determinations for these oxidation states in other TMO cathode materials.^53,54^ For Mn, on the other hand, the average Mn–O distance remains essentially constant between the two pure components at 1.90(2) Å, a value that aligns with earlier EXAFS findings on similar Mn-containing cathodes.^54,55^ Note that a 21 pm reduction of the ionic radius has been reported for Ni^2+^ to Ni^4+^ transition, while going from Mn^3+^ to Mn^4+^ implies only a 5 pm contraction.^56^ In addition, the uncertainties reported for the parameter determination reflect only the statistical errors from the EXAFS fitting and do not include any contribution from MCR analysis. Nevertheless, the high sensitivity of the EXAFS region to interatomic distances, combined with the pronounced change in the Ni–O bond lengths and the essentially unchanged Mn–O distances, as well as the negligible shift in the onset energy of the Mn K-edge spectra for the pure components (Figure 4b), all corroborate the observed trends and support little Mn participation in the redox activity. Both an invariant Mn oxidation state and a Mn^3+^/Mn^4+^ redox activity were previously detected by XAS on other TMO cathodes, thus highlighting that Mn participation in charge compensation is critically dependent upon the specific material formulation.^40,45,46,53–55^

**Figure 5:**
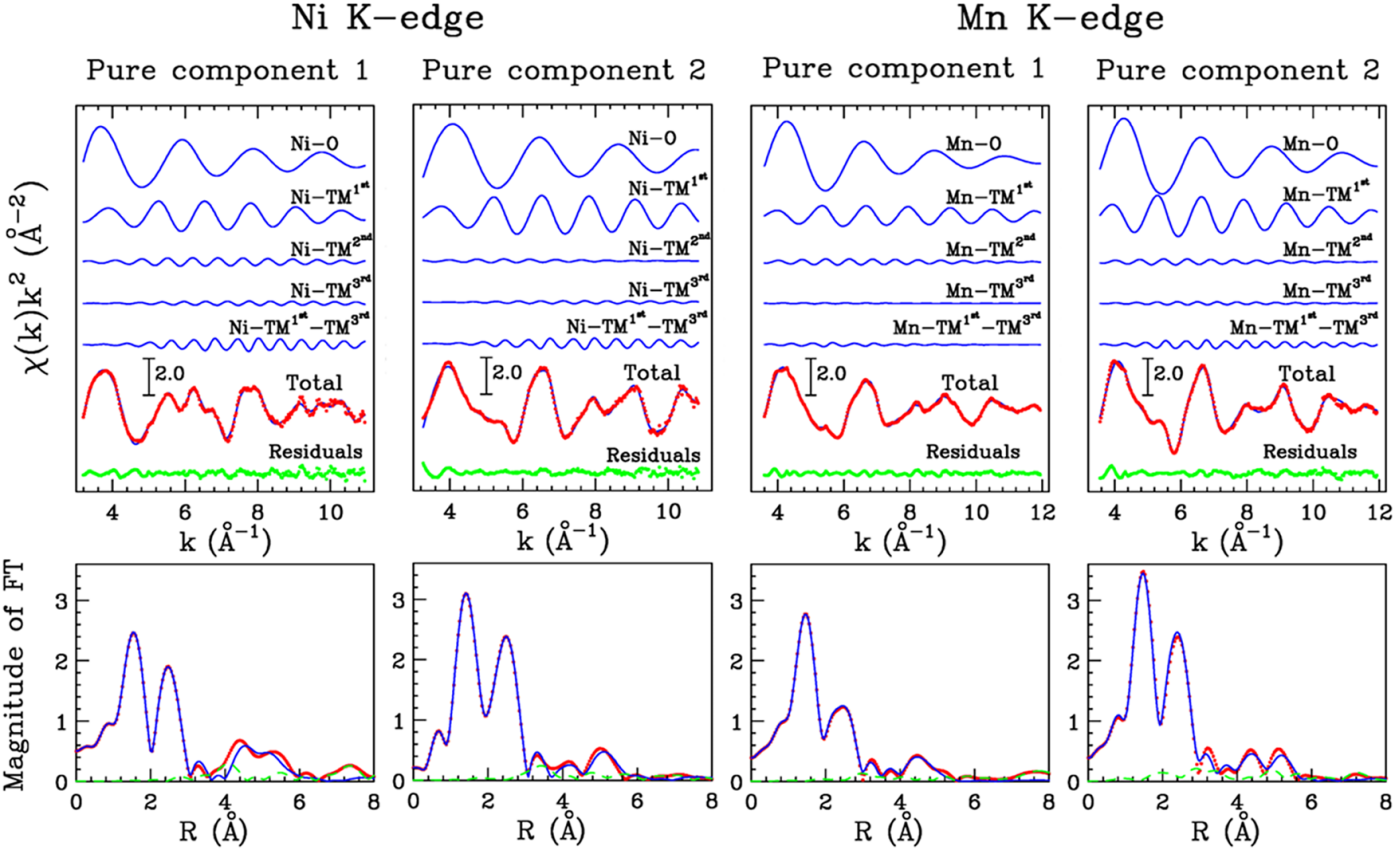
EXAFS data analysis of the Ni and Mn K-edge MCR-derived pure spectral components. Top panels: from the top to bottom, theoretical signals and total theoretical contribution (blue lines) compared with the experimental data (red dots) and the resulting residuals (green dots). Lower panels: nonphase shift corrected FT of the best-fit theoretical signal (blue line), of the experimental data (red dots), and of the resulting residuals (green dashed line). The FT was calculated in the 3.2–10.9 and 3.6–11.9 Å^–1^ *k*-range for the Ni and Mn K-edge spectra, respectively.

**Table 2:** Structural Parameters for the Ni­(Mn)–O and Ni­(Mn)–TM^1st^ Two-Body Distributions Obtained from the EXAFS Data Analysis of the Ni and Mn K-edge MCR Analysis-Derived Pure Spectral Components^a^

		*N*	*R* (Å)	σ^2^ (Å^2^)	β
Ni K-edge					
Pure component 1	Ni–O	5.5(5)	2.04(2)	0.002(2)	0.0(1)
	Ni–TM^first^	4.0(6)	2.90(3)	0.005(3)	0.0(2)
Pure component 2	Ni–O	5.5(5)	1.89(2)	0.001(3)	0.7(1)
	Ni–TM^first^	4.3(6)	2.87(3)	0.003(3)	0.7(2)
Mn K-edge					
Pure component 1	Mn–O	6.0(5)	1.90(2)	0.005(2)	0.1(1)
	Mn–TM^first^	2.0(6)	2.87(3)	0.005(3)	0.7(2)
Pure component 2	Mn–O	6.0(5)	1.90(2)	0.003(2)	0.1(1)
	Mn–TM^first^	5.0(6)	2.89(3)	0.008(3)	0.7(2)

a *N* is the coordination number, *R* the average distance, σ^2^ the Debye–Waller factor, and β the asymmetry index. Standard deviation in parentheses.

### DFT Modeling of the LRLO Lattices: Diffuse Oxygen Redox Activity and MnO_6_ Distortions

We further studied the electronic structure of the LRLO lattice by DFT+U calculations on the FL-LRLO (Li_1.28_Ni_0.15_Mn_0.57_O_2_) and PL-LRLO (Li_0.71_Ni_0.15_Mn_0.57_O_2_) stoichiometries. The average magnetic moments obtained for the Ni, Mn, and O sites are 0.825, 3.178, and −0.294 μ_B_ for FL-LRLO, confirming the mean +3 (low spin), +4, and −2 oxidation states, respectively,^57,58^ as already estimated based on stoichiometric considerations and evidenced, in the case of Mn and Ni, by XAS analysis. On the other hand, the magnetic moments in the PL-LRLO are 0.085, 3.086, and −0.247 μ_B_ for the Ni, Mn, and O sites. These values suggest a + 4 oxidation state for both Ni and Mn, in line with XAS data, whereas the slight increase of oxygen average magnetic moment compared to the FL-LRLO is consistent with an O^2–^/O^–^ redox activity.

The atom-projected density of states (PDOS) for the FL-LRLO and PL-LRLO phases are shown in Figure S12. The pristine FL-LRLO has a 0-bandgap character, in line with other Co-free LRLOs,^58^ whereas the PL-LRLO has a metallic character. With PL-LRLO being an electron-deficient, Li-poor variant of the original LRLO lattice, all valence and conduction bands in the PL-LRLO are shifted to higher energies, thus crossing the Fermi level compared to the PDOS of FL-LRLO. Moreover, close to the Fermi level, there is an increase in the hybridization of Ni and Mn d-states with the p-states of the oxygen anions in the PL-LRLO, whereas in the FL-LRLO, Ni states prevail in the proximity of the Fermi level. Overall, the comparison of the electronic DOS of FL-LRLO and PL-LRLO supports the redox activity of Ni and O. Interestingly, the oxygen-projected PDOS is smeared in an extended energy range in the PL-LRLO, also crossing the Fermi energy. This shape suggests, besides the Ni oxidation, electrons to be removed from O bands delocalized across the entire O sublattice rather than being localized on specific oxygen atomic sites. In this view, the O-redox reaction in this specific p-doped Co-free stoichiometry appears not to involve a localized O^2–/^O^–^ redox couple but, more likely, a diffuse electron-poor anion sublattice. This redox mechanism can explain the near absence of molecular oxygen evolution upon charge observed by OEMS (Figure 2d), as the formation of two proximal peroxide centers able to disproportionate to oxide and O_2_ is unlikely. Also note that Mn-derived states intersecting the Fermi level upon delithiation (Figure S12) seem contradictory to the negligible Mn participation in redox activity. A plausible interpretation is that the Mn PDOS might indicate partial electronic hybridization or interaction involving Mn orbitals that, while not necessarily constituting a formal change in its average oxidation state, imply a localized charge redistribution within the lattice. Such electronic involvement, even if localized or transient, could significantly impact the local coordination environment and symmetry around Mn.

Returning to the impact of delithiation on the local metal coordination shells, the evolution of the pair distribution functions (PDFs) for the TM-O distances (M = Ni, Mn) is shown in Figure 6b together with a schematic representation of the optimized FL-LRLO and PL-LRLO supercells (Figure 6a). The contraction of Ni–O distances from FL-LRLO to PL-LRLO (Figure 6b) aligns with the Ni^3+^/Ni^4+^ redox and XRD and XAS results discussed above. Also consistent with the experimental results above, Mn–O distances are less affected by deintercalation. To unveil the possible subtle structural changes around the Mn centers upon deintercalation, combined distribution functions (CDFs) between Mn–O distances and the O–Mn–O angular distribution have been calculated (Figure 6c).^59^ In the pristine state, two areas of high probability can be seen for a broad range of distances between 1.8–2.2 Å, with angular distributions around ∼90° and 180°. This distribution is the typical fingerprint of octahedral coordination.^59–61^ The PL-LRLO structure has a different angular distribution, as angles between 90° and 180° are detected, and angles around 90° are more broadly dispersed. This indicates a distortion of the MnO_6_ octahedra during delithiation, which may drive the irreversible structural rearrangement detected for Mn upon cycling by *operando* XAS.

**Figure 6:**
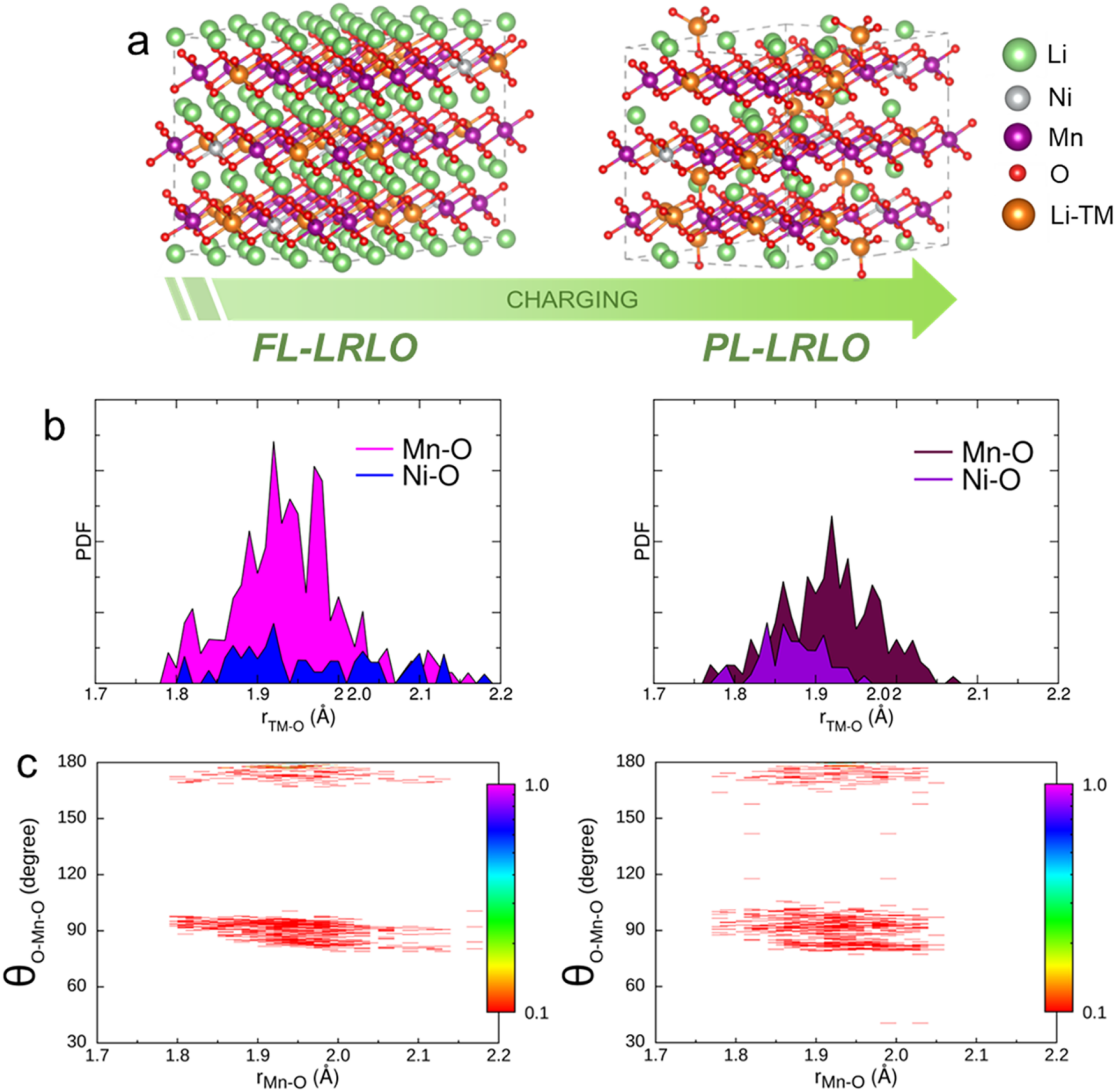
(a) DFT + *U* optimized geometries and volume structures of the pristine FL-LRLO (left panel) and delithiated PL-LRLO (right panel) supercells. The atoms are shown according to the color code reported on the right, where Li-TM denotes the Li atoms embedded in the TM layers. (b) PDFs calculated for the Ni–O and Mn–O pairs, and (c) CDFs between Mn–O distances and O–Mn–O angles calculated from the optimized structures. The color boxes on the right indicate the probability on a logarithmic scale of finding the observed atoms at the corresponding distances and angles, normalized to the maximum probability. Left panels: FL-LRLO, right panels: PL-LRLO structure.

To validate this hypothesis, theoretical Mn K-edge XANES spectra have been calculated from the DFT + *U* optimized structures. Experimental and calculated spectra are compared in Figure S11. The computed spectra nicely predict the slight but evident differences between the MCR-derived spectra of pure components 1 and 2, corresponding to the pristine and delithiated states, respectively. These are the relative intensities of the pre-edge transition at ∼6550 eV, the energy shift of the white-line position, and the lower bump around ∼6593 eV in the delithiated state. Also, the increased intensity of the pre-edge peaks in the delithiated LRLO is predicted, albeit shifted toward higher energies in the theoretical spectra (inset in Figure S11). The latter is typical for the FDM theory due to approximations in the exchange-correlation potential.^62^ The theoretical XANES calculations, therefore, validate the structures obtained at the DFT + *U* level for the pristine and delithiated LRLO. This combined result strongly supports the interpretation that distortions in the centrosymmetric MnO_6_ octahedra triggered by the sequential Ni- and O-redox activity are the predominant changes affecting Mn during the formation, and that this structural rearrangement is the key to the irreversible activation of the LRLO cathode.

## Conclusions

This study provides a comprehensive clarification of the structural and electronic transformations induced by electrochemical formation in the Co-free, Ni-poor LRLO positive electrode material Li_1.28_Ni_0.15_Mn_0.57_O_2_. A three-stage formation with increasing rates and hold steps at 4.8 V was used to enhance the performance after formation. Parasitic chemistry levels off during formation, releasing CO_2_ and minor O_2_. In parallel, the electrode/electrolyte interface consolidates, as seen in the impedance response. However, formation leads to remarkable changes in the lattice composition and structure. The lithiated lattice composition stabilizes at 0.88 Li in the formula unit. This change comes with a permanent rearrangement of the crystal structure as seen by *operando* XRD. Activation most prominently compresses the layer spacing along the *c*-axis and increases structural breathing, reversibly accommodating more significant variations of the cell parameters than in initial cycles.

Changes to structure, composition, and electronics have been probed in detail using *operando* XAS and DFT+U modeling. Chemometric analysis of the XAS data demonstrates fully reversible Ni^2+^/Ni^4+^ redox activity despite the presence of Ni^3+^ in the pristine material. DFT + *U* suggests, after the complete oxidation to Ni^4+^, a delocalized O-redox smeared through the anionic sublattice. This diffuse charge removal differs from mechanisms involving localized peroxide centers, thus preventing detrimental molecular oxygen evolution and explaining the near absence of O_2_ release.

XAS, DFT+U, and simulated XANES consistently indicate that the Mn centers do not participate in the redox activity and persist in the Mn^4+^ state over cell cycling. On the other hand, the internal lattice stress induced by sequential Ni- and O-redox is dissipated through structural distortions of the MnO_6_ octahedra. These changes to the Mn environment accumulate irreversibly during the formation and are key to activating this LRLO cathode, enabling superior electrochemical performance.

The results obtained provide a mechanistic foundation for understanding the high reversible capacity of Co-free, Ni-poor LRLOs and help establish guiding principles for the design of next-generation, high-capacity cathode materials with improved environmental sustainability.

## Supplementary Material



## Notes

Additional experimental and methodological details related to the electrochemical, XRD, XAS, and DFT studies, together with complementary results (PDF)
