# Supplementary material for: Structural Rearrangements of a Cobalt-Free Lithium-Rich Layered Oxide Cathode during Formation
Source: ACS Appl Energy Mater. 2025 Dec 21;9(1):686–97. doi: 10.1021/acsaem.5c03511 (PMC12801421; doi:10.1021/acsaem.5c03511)
Supplement: Supplementary file 1 [file ae5c03511_si_001.pdf]

Structural Rearrangements of a Cobalt-Free Lithium-Rich Layered Oxide Cathode During Formation

Matteo Busato[a,b], Mariarosaria Tuccillo[a,b,c], Arcangelo Celeste[a,b,c], Alessandro Tofoni[a,b], Laura Silvestri[c], Paola D'Angelo[a,b], Stefan A. Freunberger[b,d,*], and Sergio Brutti[a,b,e,f,*]

[a]Department of Chemistry, Sapienza University of Rome, P.le Aldo Moro 5, 00185, Rome, Italy

[b]ALISTORE European Research Institute (ALISTORE ERI), Hub de l'Energie, 15 Rue Baudelocque, 80039, Amiens, France

[c]Department of Energy Technologies and Renewable Sources, ENEA, C.R. Casaccia via Anguillarese 301, 00123, Rome, Italy

[d]Institute of Science and Technology Austria (ISTA), Am Campus 1, 3400 Klosterneuburg, Austria

[e]GISEL-Centro di Riferimento Nazionale per i Sistemi di Accumulo Elettrochimico di Energia, 50121, Florence, Italy

[f]Istituto dei Sistemi Complessi, Consiglio Nazionale delle Ricerche, P.le Aldo Moro 5, 00185, Rome, Italy

Keywords: lithium-rich layered oxides, Li-ion battery, cathode, Co-free, electrochemical performance, DFT, X-ray absorption spectroscopy

*Corresponding author
e-mail address: stefan.freunberger@ist.ac.at, sergio.brutti@uniroma1.it

Supporting Information

Pages: S1-S15

Figures: S1-S12

Tables: S1-S2

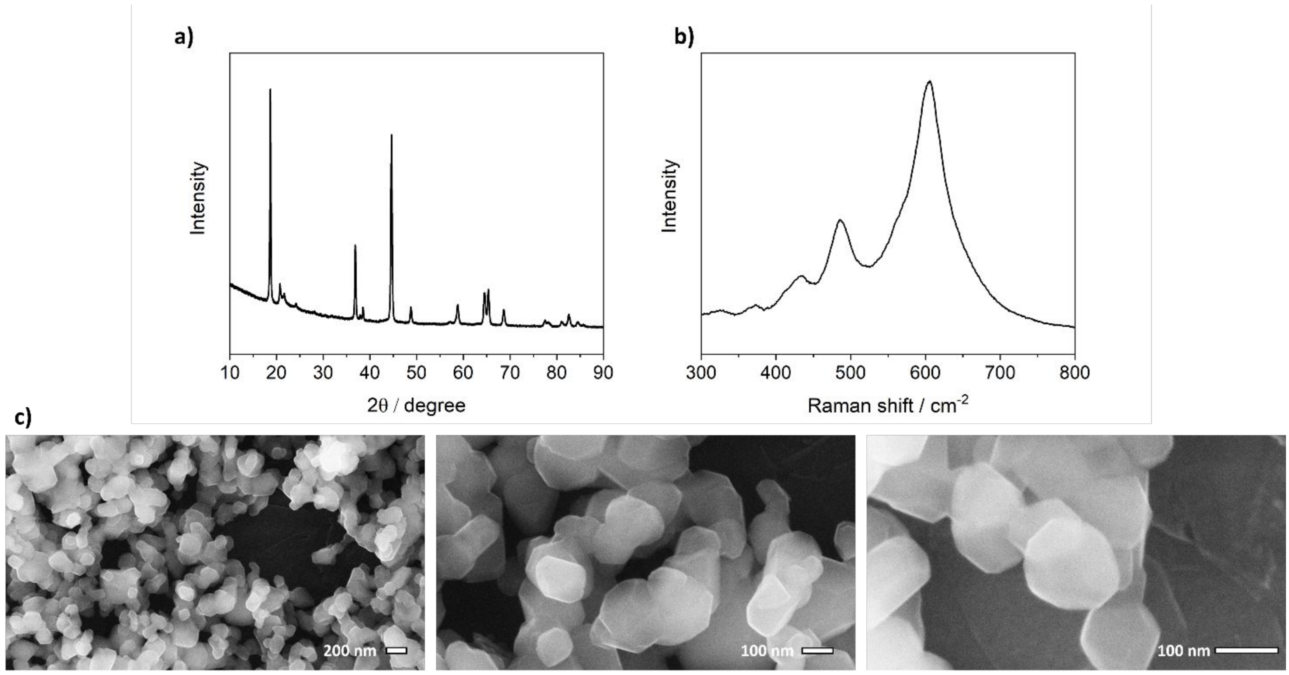

Figure S1. Chemical-physical characterisation of $Li_{1.28}Ni_{0.15}Mn_{0.57}O_2$: a) X-ray diffraction (XRD) pattern, b) Raman spectrum, and c) scanning electron microscopy micrographs at different magnifications.

Electrochemical Characterization. Solutions for the electrochemical Li^+ titration (ELT) tests have been prepared by dissolving a weighted amount of the LRLO materials in a 1.0 M H_2SO_4 solution and adding NH_3 to keep the solution at pH 7.0 for 24 h. The obtained solutions have been quantitatively transferred and diluted in a volume-calibrated task using de-ionized water to a target concentration of approximately 1 – 10 ppm. The concentration of free Li^+ ions in the final solution has been measured using a pre-calibrated Mettler Toledo DX2017-Li ion-selective electrode.

Inductively coupled plasma optical emission spectroscopy (ICP-OES) allowed us to measure the compositions of all metals in the LRLO (Li, Mn, and Ni). Samples were dissolved using aqua regia (Merck Millipore Nitric acid 65 % - EMD Millipore Hydrochloric acid 37 %) at 80 °C under magnetic stirring. After dissolution, the samples were filtered to remove the undissolved solid (as carbon) and diluted using 0.5 M HNO_3.

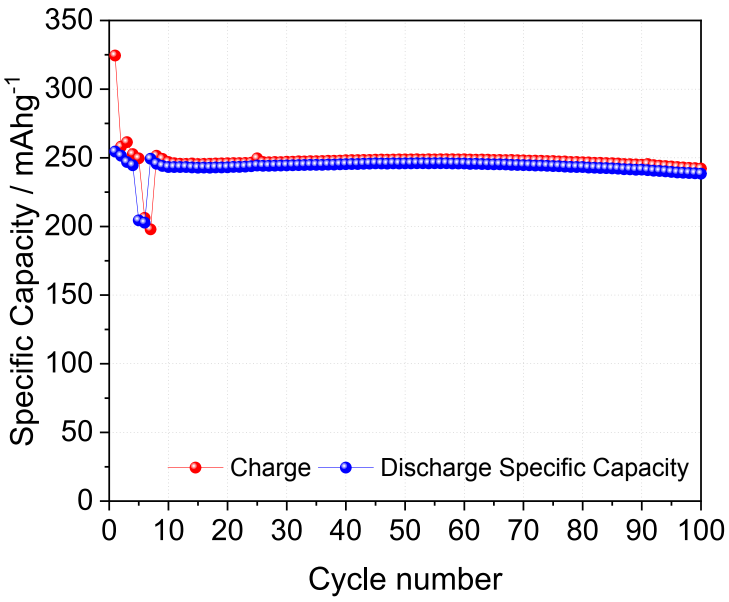

Figure S2. Specific capacity vs cycle number during galvanostatic cycling at C/10 between 2–4.8 V of the activated LRLO cathode in a lithium cell.

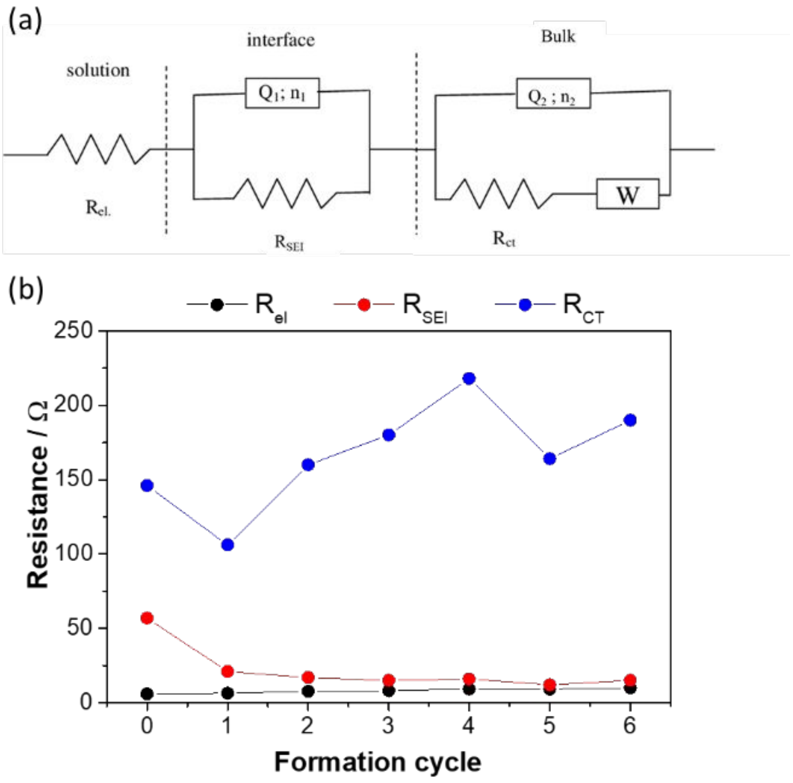

Figure S3. a) Equivalent circuit model used to evaluate EIS spectra recorded at endpoints in the formation procedure, b) evolution of the electrolyte resistance (R_{el}), solid-electrolyte interphase (SEI) resistance (R_{SEI}), and charge transfer resistance (R_{CT}) during formation.

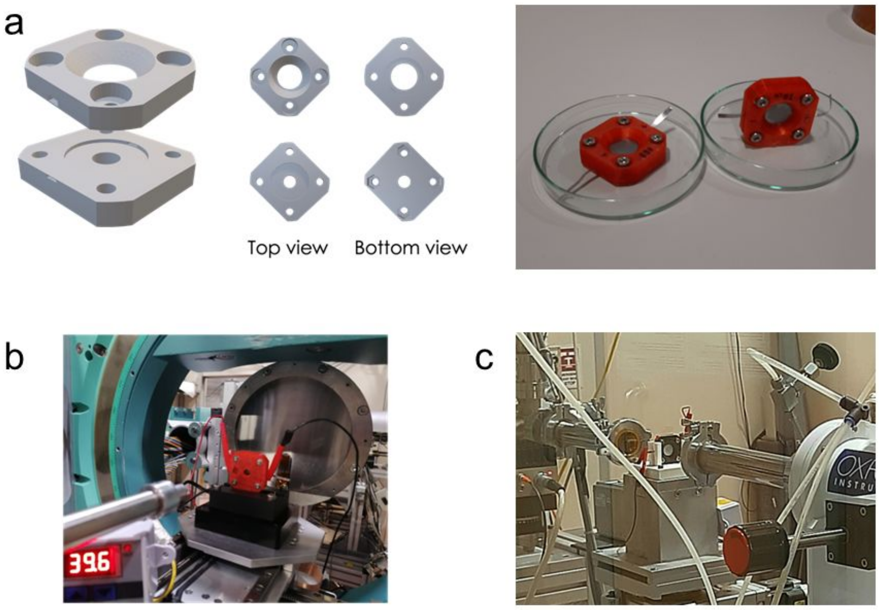

Figure S4. a) 3D-printed cells in polylactic acid with beryllium windows used for the *operando* XRD and X-ray absorption (XAS) measurements on the formation cycles of the LRLO cathode material. Experimental setups at the b) MCX and c) XAFS beamlines of Elettra-Sincrotrone Trieste, where the XRD and XAS measurements have been performed, respectively.

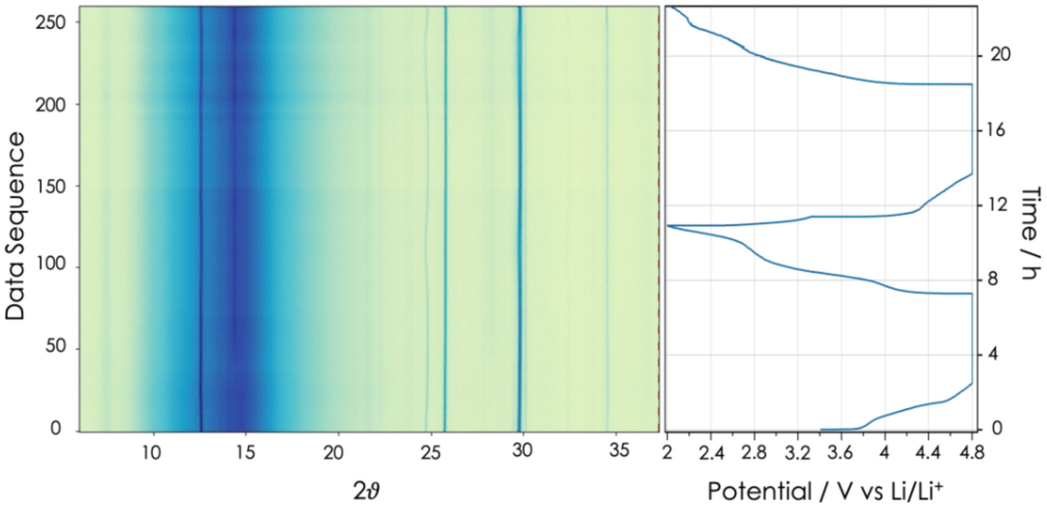

Figure S5. *Operando* XRD dataset recorded starting from a pristine LRLO electrode (XRD acquisition time of 20 seconds) during two consecutive galvanostatic cycles at C/10 between 2.0 and 4.8 V each with a potentiostatic hold at 4.8 V (end of charge) with a current cutoff of C/20.

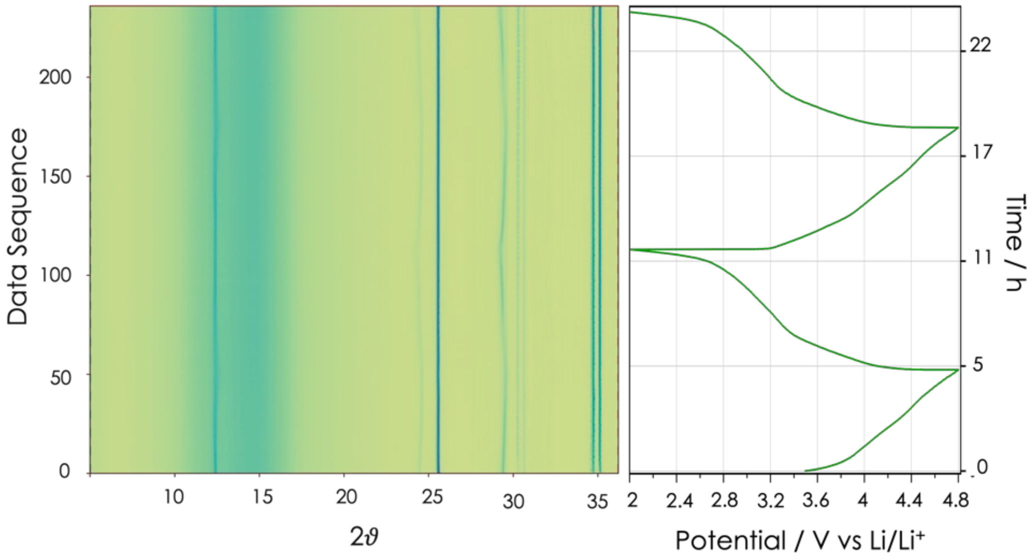

Figure S6. *The Operando XRD dataset was* recorded starting from an LRLO electrode after a full activation (XRD acquisition time of 20 seconds) during two consecutive galvanostatic cycles at C/10 between 2.0 and 4.8 V.

Decomposition of the *operando* XAS data into the spectra and fractional concentrations of key components. Time-resolved spectroscopical measurements of chemical processes yield a series of spectra that can be positioned in a matrix $\boldsymbol{D}$, where its columns are the spectra measured at time *t*. According to Lambert-Beer's law, the measured signal at any given time can be expressed as a linear combination of the signal belonging to a number *N* of "pure" and independent components, weighed by their fractional concentration.[1] In this work, the decomposition of the experimental data into the spectra associated with the key species and their relative concentrations was performed with the PyFitit code,[1] a software that uses an algorithm belonging to the multivariate curve resolution (MCR) family.

The starting point is the Singular Value Decomposition (SVD) equation:

$$\boldsymbol{D} = \boldsymbol{U} \cdot \boldsymbol{\Sigma} \cdot \boldsymbol{V} + \boldsymbol{E} \qquad \text{(S1)}$$

where the product $\boldsymbol{U} \cdot \boldsymbol{\Sigma}$ contains, on its *N* columns, a set of values that may be associated with the normalized absorption coefficients, $\boldsymbol{\Sigma}$ is a diagonal matrix known as the singular values term, whose elements are sorted in decreasing order, while $\boldsymbol{V}$ can be interpreted as the concentration matrix associated with the *N*-selected components. Lastly, the error matrix $\boldsymbol{E}$ represents the lack of fit between the experimental data matrix $\boldsymbol{D}$ and the reconstructed matrix $\boldsymbol{U} \cdot \boldsymbol{\Sigma} \cdot \boldsymbol{V}$. The SVD decomposition depends on the correct estimation of the number of components *N* present in the experimental spectral matrix. To this end, in this work, we evaluated *N* by performing a scree plot test, whose results are shown in **Figure S7**. In the scree plot, the singular values relative to each

principal component are plotted against the number of pure components. The presence of an elbow in such a curve separates the signal and noise-related components.

At this stage, all matrices in eq. S1 are solely mathematical solutions to the decomposition problem without physical-chemical meaning. Once N is established, the approach implemented by the PyFitIt code requires the introduction of a transformation N x N matrix $\boldsymbol{T}$ in eq. S1, using the relation $\boldsymbol{I} = \boldsymbol{T} \cdot \boldsymbol{T}^{-1}$:

$$\boldsymbol{D} = \boldsymbol{U} \cdot \boldsymbol{\Sigma} \cdot \boldsymbol{T} \cdot \boldsymbol{T}^{-1} \cdot \boldsymbol{V} + \boldsymbol{E} \qquad \text{(S2)}$$

where the spectra belonging to the key species are given by $\boldsymbol{S} = \boldsymbol{U} \cdot \boldsymbol{\Sigma} \cdot \boldsymbol{T}$ and their concentration profiles by $\boldsymbol{C} = \boldsymbol{T}^{-1} \cdot \boldsymbol{V}$. Subsequently, the matrix elements T_{ij} of matrix $\boldsymbol{T}$ are manually selected by sliders to achieve $\boldsymbol{S}$ and $\boldsymbol{C}$, which are chemically and physically interpretable. Once these are achieved, one can finally write:

$$\boldsymbol{D} = \boldsymbol{S} \cdot \boldsymbol{C} + \boldsymbol{E} \qquad \text{(S3)}$$

The unknown number of T_{ij} elements of $\boldsymbol{T}$ is, in principle, equal to N^2.

In the case of the Mn K-edge data set, the X-ray absorption near edge structure (XANES) spectrum collected on the pristine electrode was constrained to coincide with the first extracted spectral component. This operation, together with the imposed normalization of each extracted component, allows the reduction of the number of unknown T_{ij} elements from N^2 to $N^2 - N$.[1] Differently, only the normalization constraint was applied to the Ni K-edge data set due to the mixed Ni^{2+}/Ni^{4+} composition of the pristine electrode (see the main text).

In the case of the Ni K-edge data set, the MCR analysis was carried out by considering either two or three independent components. For the former case, a 2 x 2 matrix $\boldsymbol{T}_{Ni}$ was defined containing four elements, and the solution for the decomposition presented in eq. S3 was obtained using the following values:

$$\boldsymbol{T}_{Ni} = \begin{pmatrix} -0.19 & -0.19 \\ -0.30 & 0.45 \end{pmatrix} \qquad \text{(S4)}$$

while with three components:

$$\boldsymbol{T}_{Ni} = \begin{pmatrix} -0.19 & -0.19 & -0.19 \\ -0.30 & 0.30 & -0.45 \\ 0.00 & 0.00 & 0.00 \end{pmatrix} \qquad \text{(S5)}$$

In the case of the Mn K-edge data set, with two independent components, the following transformation matrix was obtained:

$$\boldsymbol{T}_{Mn} = \begin{pmatrix} -0.18 & -0.18 \\ -0.41 & 0.30 \end{pmatrix} \tag{S6}$$

The energy range was 8200–8800 eV and 6400–7100 eV for Ni and Mn K-edge, respectively. Solely non-negative XAS spectral and concentration profiles were accepted as solutions in all cases.

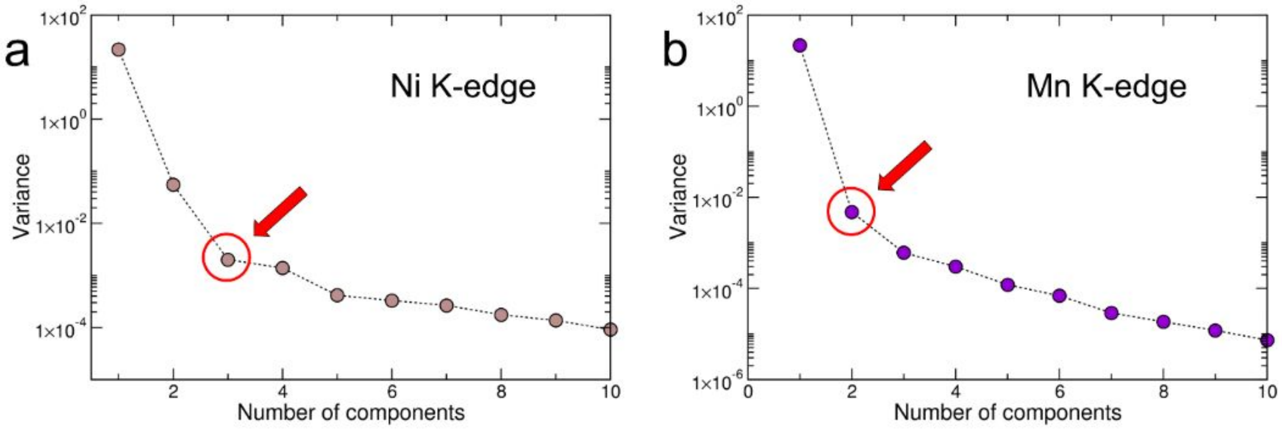

Figure S7. Scree plot statistical analysis of the a) Ni and b) Mn K-edge *operando* XAS data for the first ten components. The arrow indicates the presence of N = 3 and N = 2 components for Ni and Mn, respectively.

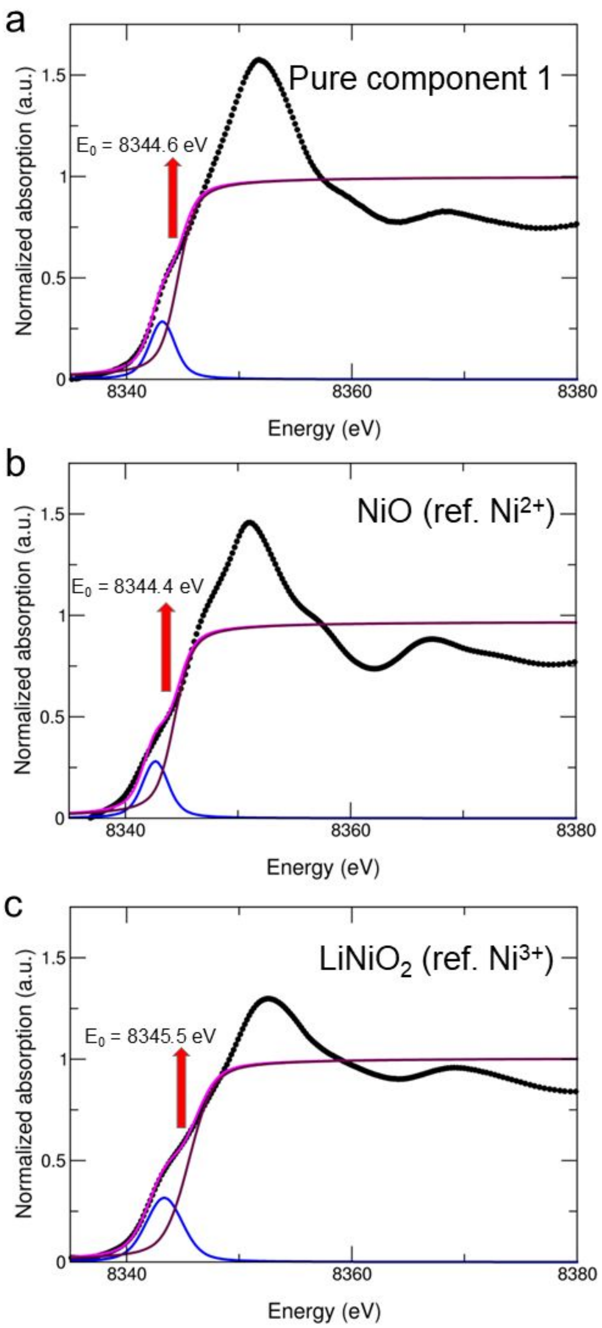

Figure S8. Fitting of the edge energy region of Ni K-edge XANES data of the a) MCR analysis-derived pure component 1, b) NiO, and c) $LiNiO_2$ references (black dots) with a Lorentzian function (blue line) accounting for the pre-edge contribution and an arctangent function (maroon line) for the K-edge transition, together with the sum of the two theoretical curves (magenta line). The obtained E_0 position is reported for the sake of clarity.

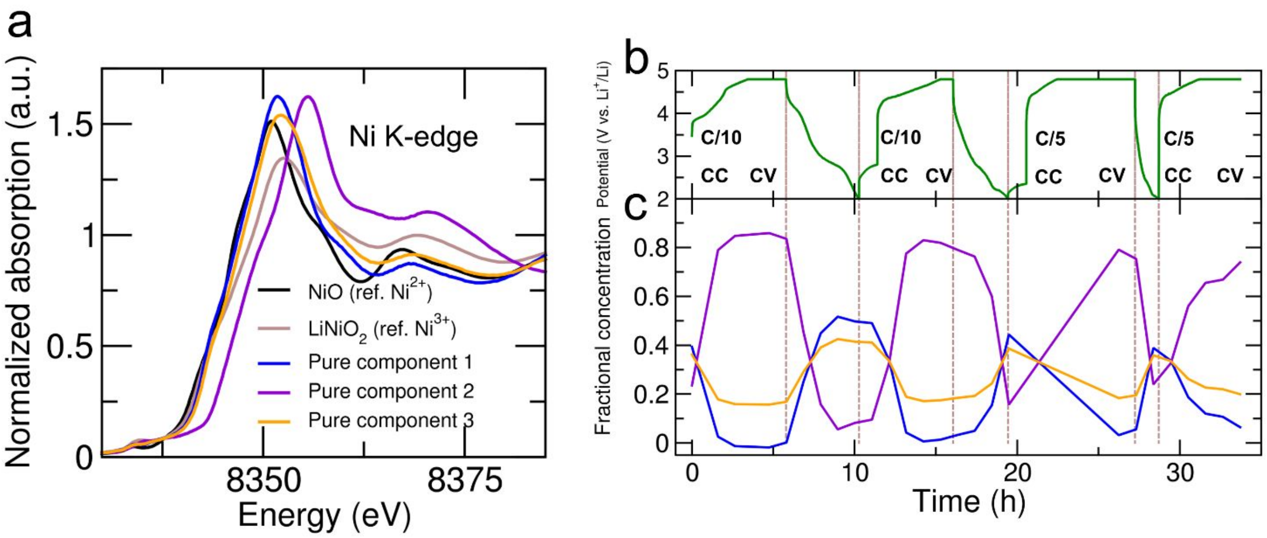

Figure S9. a) XANES spectra, b) potential, and c) concentration profiles for Ni K-edge derived from the MCR analysis considering three pure components. Vertical lines are added to indicate the different charge states. CC and CV indicated constant current and voltage phases during charging.

EXAFS data analysis. The EXAFS region of the MCR reconstructed absorption spectra of the pure components for the Ni and Mn K-edge data sets was analyzed with the GNXAS code.[2,3] Amplitudes and phase shifts have been calculated from clusters with fixed geometry within the muffin-tin (MT) approximation. The employed clusters were obtained by cutting a 6 Å radius portion around a representative Ni or Mn center from the fully lithiated LRLO optimized structure obtained by the Hubbard-modified density functional theory (DFT+U) simulations (*vide infra*). The MT radii were chosen to produce a ~20 % overlap of the adjacent MT spheres and were 1.60, 1.40, 0.90, and 0.20 Å for Ni, Mn, O, and Li, respectively. Advanced models for the exchange-correlation self-energy in the Hedin–Lundqvist scheme accounted for the photoelectron inelastic losses in the final state.[4]

In the GNXAS approach, theoretical signals associated with *n*-body distribution functions are calculated following the multiple-scattering (MS) theory and summed to reconstruct the total theoretical contribution. Theoretical Ni-O and Mn-O single-scattering (SS) signals accounting for the first-shell oxygen atoms have been calculated for the Ni and Mn K-edge spectra, respectively. In addition, theoretical signals have been included to account for farther-shell Ni and Mn scattering atoms. Similar atomic numbers made it difficult to discriminate between these scattering centers, and thus, the signals are indicated as either Ni-TM or Mn-TM (TM = transition metal). In particular, Ni(Mn)-TM^{1st} and Ni-TM^{3rd} SS paths are referred to as the TMs belonging to the same stacked layer in the LRLO structure at increasing distances. A three-body MS path with a 180° bond angle was also included (Ni(Mn)-TM^{1st}-TM^{3rd}). In addition, an SS path has been added to consider the TM scattering centers belonging to different TM layers (Ni(Mn)-TM^{2nd}). A pictorial representation of these paths is given in **Figure S10**.

Each two-body distribution has been modelled as a Γ-like function depending on four structural parameters, namely the coordination number N, the average distance R, the Debye-Waller factor σ^2, and the asymmetry index β, which have been optimized during the fitting procedure to obtain the best agreement with the experimental data. Least-squares minimizations have been carried out on the raw data directly, without preliminary background subtraction and Fourier filtering, in the 3.2–10.9 and 3.6–11.9 $Å^{-1}$ k-range for the Ni and Mn K-edge spectra, respectively. Additional non-structural parameters have been optimized, namely E_0, the K-edge ionization energy, and the energy position and amplitude of the KM_1 and $KM_{2,3}$ double-electron excitation channels. The amplitude reduction factor S_0^2 was constrained between 0.85 and 1.00.

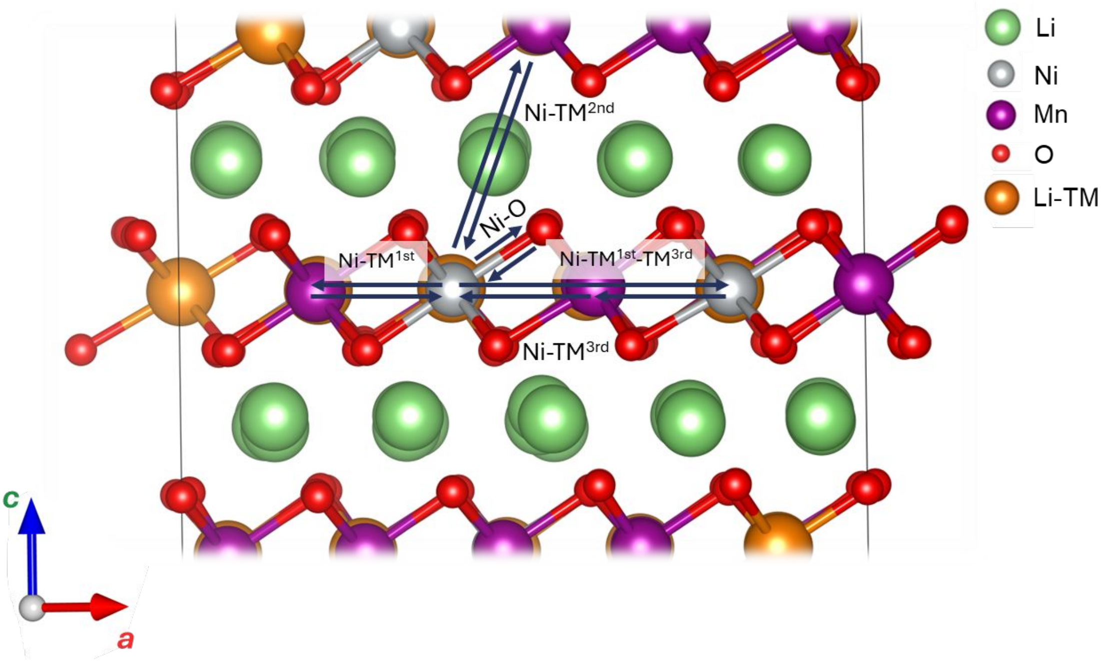

Figure S10. Pictorial representation of the SS and MS paths considered in the EXAFS analysis. The example shown refers to the Ni absorber. Atoms shown according to the color code reported on the top-right, where Li-TM denotes the Li atoms embedded in the TM layers.

Table S1. Structural parameters for the Ni-TM^{2nd} and Ni-TM^{3rd} two-body SS distributions obtained from the EXAFS data analysis of the Ni and Mn K-edge MCR analysis-derived pure spectral components.[a]

		N	*R* (Å)	σ^2 (Å^2)	β
**Ni K-edge**					
Pure component 1	Ni-TM^{2nd}	6.0(6)	5.04(3)	0.009(3)	0.7(2)
	Ni-TM^{3rd}	4.0(6)	5.73(3)	0.004(3)	0.0(2)
Pure component 2	Ni-TM^{2nd}	5.0(6)	4.97(3)	0.019(3)	0.1(2)
	Ni-TM^{3rd}	4.3(6)	5.70(3)	0.006(3)	0.0(2)
**Mn K-edge**					
Pure component 1	Mn-TM^{2nd}	4.0(6)	5.07(3)	0.012(3)	0.7(2)
	Mn-TM^{3rd}	2.0(6)	5.64(3)	0.022(3)	0.0(2)
Pure component 2	Mn-TM^{2nd}	6.0(6)	5.06(3)	0.018(3)	0.7(2)
	Mn-TM^{3rd}	5.0(6)	5.76(3)	0.012(3)	0.0(2)

[a]*N* is the coordination number, *R* the average distance, σ^2 the Debye-Waller factor, and β the asymmetry index. Standard deviation in parentheses.

Theoretical XANES simulation. Advanced theoretical calculations of Mn K-edge XANES spectra have been carried out using the finite differences method near-edge structure (FDMNES) program. This DFT code can calculate XAS spectra within the entire MS and finite differences method (FDM) theoretical frameworks.[5] This allows, in principle, the simulation of the photoelectron wavefunction beyond 100 eV above the absorption edge, avoiding the MT approximation used in many common MS theory-based codes.[5,6] In the FDM framework, the unit cell-normalized cross-section $\sigma(\omega)$ is calculated as:

$$\sigma(\omega) = 4\pi^2\alpha\hbar\omega\sum_j \sum_{f,g} |\Psi_f|\Theta|\Psi_g^{(j)}|^2\delta(\hbar\omega - (E - E_g^{(j)})) \qquad \text{(S7)}$$

where $\hbar\omega$ is the photon energy, α the fine structure constant, E_g and *E* the energies of the ground-states $\Psi_g^{(j)}$ and Ψ_f, respectively. At the same time, the summation over *j* includes the contribution of all the atoms in the unit cells possessing index *j*.[7] The electron-photon interaction is treated classically employing the Θ operator, neglecting the magnetic part of the electromagnetic field and describing its electric portion with the first two terms of the multipolar expansion, which correspond to the electric dipole and quadrupole excitations:

$$\Theta = \boldsymbol{\varepsilon}\cdot\boldsymbol{r}(1 + \frac{i}{2}\boldsymbol{k}\cdot\boldsymbol{r}) \qquad \text{(S8)}$$

where ***r*** is the relative position from the photo absorber, ε is the photon polarization, and *k* is the photon wavevector. In all calculations, the Schrödinger-like equation is solved self-consistently to find the final states where there is a transition.[7] In a post-processing step, the calculated cross-sections were convoluted by an energy-dependent Lorentzian function of variable width Γ_{tot} to allow direct comparison with the experimental XANES spectra. Γ_{tot} is expressed as the sum of a tabulated core-hole lifetime width Γ_{hole} and an arctangent function $\Gamma(\omega)$ defined as follows:

$$\Gamma_{\text{tot}}(E-E_F) = \Gamma_{\text{hole}} + \Gamma(\omega) = \Gamma_{\text{hole}} + \Gamma_{\text{max}}\left(\frac{1}{2} + \frac{1}{\pi}\arctan\left(\frac{\pi\Gamma_{\text{max}}}{3E_l}\left(\frac{E-E_F}{E_{ctr}} - \frac{E_{ctr}^2}{(E-E_F)^2}\right)\right)\right) \quad \text{(S9)}$$

where *E* is the energy scale of the Mn K-edge XANES spectrum, E_F the Fermi energy, and Γ_{max}, E_l, and E_{ctr} are empirical parameters representing the final-state width, width of the arctangent function, and its center, respectively. In addition, convolution with a Gaussian broadening of constant width can be applied to account for the experimental resolution.[8]

XANES spectra were calculated for the pristine and partially de-lithiated LRLO structures optimized by DFT+U simulations (*vide infra*). The calculations have been performed by randomly selecting seven Mn centers for both the pristine and charged states. Calculations in full MS and FDM theory have been then performed for structures including all atoms within 6 Å from each of the selected photo absorbers. We remark that theoretical XANES spectra, such as those computed by FDMNES, exhibit an energy shift with respect to the experimental data due to the omission of atomic and relativistic stabilization effects and systematic errors given by the employed exchange-correlation potential.[9] To correct this effect, the calculated XANES spectrum for the pristine electrode has been aligned to the onset energy of the experimental one, which is identified at the absorption unity. The theoretical spectrum of the charged state has been shifted in energy by the same amount for comparison. The applied energy shift was 3.74 eV in both cases. The tabulated Γ_{hole} value of 1.16 eV for the Mn K-edge was employed,[10] Γ_{max} was set to 1.50 eV, while E_l and E_{ctr} were kept at their default value of 30 eV.

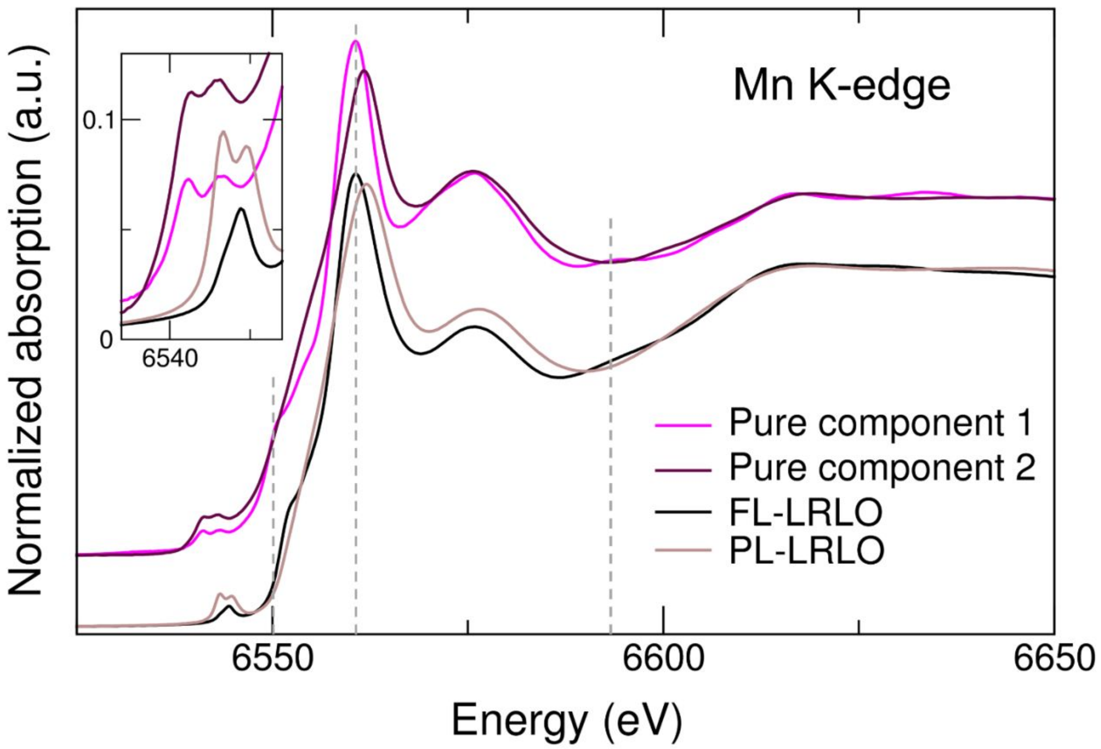

Figure S11. Comparison between the Mn K-edge XANES spectra of the MCR-derived pure components with the theoretical spectra calculated from the optimized FL-LRLO and PL-LRLO DFT+U structures. The pure component spectra are vertically shifted for the sake of clarity. Gray dashed lines serve as guides to the eye to highlight the position of the relevant spectral features. Inset: magnification of the pre-peak region corresponding to the 1s → 3d/4p transitions.

DFT+U details. The final supercells for DFT+U simulations have the following compositions: fully lithiated LRLO (FL-LRLO) $Li_{77}Ni_9Mn_{34}O_{120}$ and partially de-lithiated LRLO (PL-LRLO) $Li_{37}Ni_9Mn_{34}O_{120}$. The composition of the PL-LRLO has been assumed to match the final lithium content during the *operando* XRD test. In this way, it was possible to mimic an oxidized LRLO, where all Mn and Ni atoms are nominally in the +4 state, and a significant amount of charge density has been removed from the anion sublattice to mimic the occurrence of the O^{2-}/O^{-} redox reaction. In the reduced FL-LRLO, by postulating O^{2-} anions and Li^{+} cations, the Mn and Ni mean oxidation states are +4.0 and +3.0, respectively. In the oxidized PL-LRLO, by postulating Li^{+}, Mn^{4+}, and Ni^{4+} cations, the mean oxygen oxidation state is -1.7. In both the FL-LRLO and PL-LRLO, the disorder in the Li-TM layers has been modeled *via* the special *quasi*-random structure (SQS) approach[11] implemented in the ATAT code.[12] The same randomization protocol has also been applied to model the Li/vacancy distribution in the lithium layers in the case of PL-LRLO.

Both structural models have been optimized to the minimum energy ground states concerning cell volume, parameter ratios, and atomic positions. Despite the absence of constraints during structural relaxations, all supercells maintained hexagonal symmetry. The cell parameters of the FL-LRLO and the PL-LRLO are summarized in **Table S2** and compared to the experimental data

from the *operando* XRD test with similar compositions. Note that dispersion corrections were not included and that it is known that the omission of such corrections could systematically affect layered oxide structures by underestimating the attractive forces between the oxygen layers, typically leading to an overestimation of the c-axis interlayer spacing. Nevertheless, the DFT-simulated cell parameters agree within 2% of experimental values for both FL-LRLO and PL-LRLO stoichiometries, thus confirming the accuracy of the modelling approach.

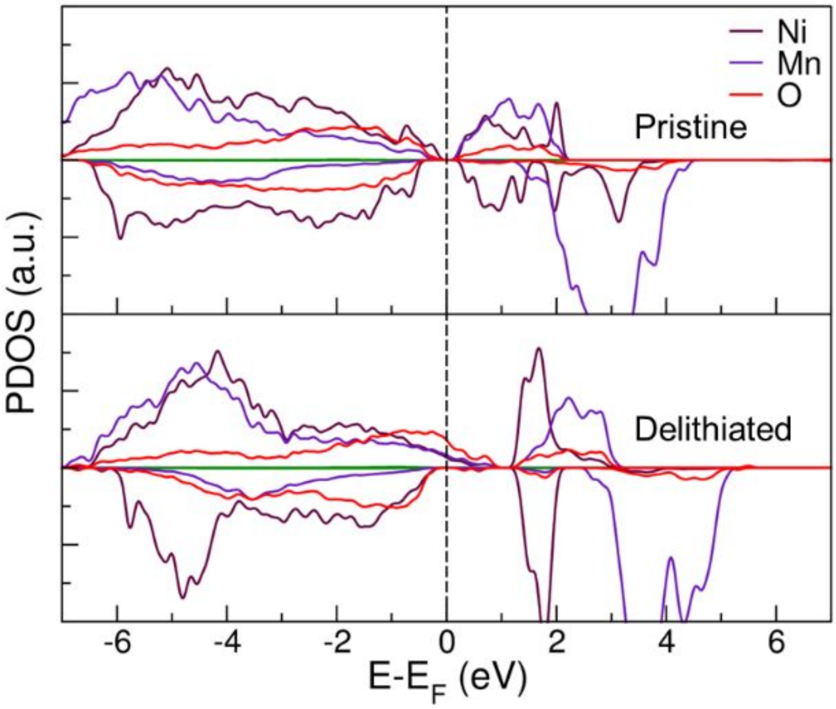

Figure S12. DFT+U atomic orbital-projected density of states (PDOSs) calculated for the Ni, Mn, and O centers on the pristine (upper panel) and partially de-lithiated (lower panel) structures of the LRLO. E_F refers to the Fermi energy.

Table S2. Cell parameters of the optimized supercells compared to experimental data (reported in parentheses) obtained by Rietveld refinement of XRD patterns with similar stoichiometries measured in the *operando* test.

	**FL-LRLO**	**PL-LRLO**	**Δ %**
a (Å)	2.892 (2.830)	2.892 (2.822)	0 % (–0.28 %)
c (Å)	14.388 (14.149)	14.392 (14.164)	+0.02 % (+0.1 %)
V (Å3/f.u.)	34.73 (32.71)	34.76 (32.56)	+0.09 % (-0.45 %)

References

1 A. Martini, S. A. Guda, A. A. Guda, G. Smolentsev, A. Algasov, O. Usoltsev, M. A. Soldatov, A. Bugaev, Y. Rusalev, C. Lamberti and A. V. Soldatov, PyFitit: The software for quantitative analysis of XANES spectra using machine-learning algorithms, *Comput. Phys. Commun.*, 2020, **250**, 107064.

2 A. Filipponi, A. Di Cicco and C. R. Natoli, X-ray-absorption spectroscopy and n-body distribution functions in condensed matter. I. Theory, *Phys. Rev. B*, 1995, **52**, 15122–15134.

3 A. Filipponi and A. Di Cicco, X-ray-absorption spectroscopy and n-body distribution functions in condensed matter. II. Data analysis and applications, *Phys. Rev. B*, 1995, **52**, 15135–15149.

4 L. Hedin and S. Lundqvist, Effects of Electron-Electron and Electron-Phonon Interactions on the One-Electron States of Solids, *Solid State Phys.*, 1970, **23**, 1–181.

5 Y. Joly, X-ray absorption near-edge structure calculations beyond the muffin-tin approximation, *Phys. Rev. B - Condens. Matter Mater. Phys.*, 2001, **63**, 1–10.

6 O. Bunău and Y. Joly, Self-consistent aspects of x-ray absorption calculations, *J. Phys. Condens. Matter*, 2009, **21**, 345501.

7 Y Joly, O Bunău, J E Lorenzo, R M Galéra, S Grenier and B Thompson, Self-consistency, spin-orbit and other advances in the FDMNES code to simulate XANES and RXD experiments, *J. Phys. Conf. Ser.*, 2009, **190**, 12007.

8 C. D. Rankine, M. M. M. Madkhali and T. J. Penfold, A Deep Neural Network for the Rapid Prediction of X-ray Absorption Spectra, *J. Phys. Chem. A*, 2020, **124**, 4263–4270.

9 S. G. Minasian, J. M. Keith, E. R. Batista, K. S. Boland, D. L. Clark, S. D. Conradson, S. A. Kozimor, R. L. Martin, D. E. Schwarz, D. K. Shuh, G. L. Wagner, M. P. Wilkerson, L. E. Wolfsberg and P. Yang, Determining Relative f and d Orbital Contributions to M–Cl Covalency in MCl_6^{2-} (M = Ti, Zr, Hf, U) and $UOCl_5^{-}$ Using Cl K-Edge X-ray Absorption Spectroscopy and Time-Dependent Density Functional Theory, *J. Am. Chem. Soc.*, 2012, **134**, 5586–5597.

10 M. O. Krause and J. H. Oliver, Natural widths of atomic *K* and *L* levels, *Kα* X-ray lines and several *KLL* Auger lines, *J. Phys. Chem. Ref. Data*, 1979, **8**, 329–338.

11 A. van de Walle, R. Sun, Q.-J. Hong and S. Kadkhodaei, Software tools for high-throughput CALPHAD from first-principles data, *Calphad*, 2017, **58**, 70–81.

12 A. van de Walle, M. Asta and G. Ceder, The alloy theoretic automated toolkit: A user guide, *Calphad*, 2002, **26**, 539–553.
